# Vertebrate Lineages Exhibit Diverse Patterns of Transposable Element Regulation and Expression across Tissues

**DOI:** 10.1093/gbe/evaa068

**Published:** 2020-04-09

**Authors:** Giulia I M Pasquesi, Blair W Perry, Mike W Vandewege, Robert P Ruggiero, Drew R Schield, Todd A Castoe

**Affiliations:** e1 Department of Biology, University of Texas at Arlington; e2 Department of Molecular, Cellular, and Developmental Biology, University of Colorado, Boulder; e3 Department of Biology, Eastern New Mexico University; e4 Department of Biology, Southeast Missouri State University; e5 Department of Ecology and Evolutionary Biology, University of Colorado, Boulder

**Keywords:** somatic transposable element expression, transposable element cellular-derived transcripts, transposable element silencing, PIWI, ovary, testis

## Abstract

Transposable elements (TEs) comprise a major fraction of vertebrate genomes, yet little is known about their expression and regulation across tissues, and how this varies across major vertebrate lineages. We present the first comparative analysis integrating TE expression and TE regulatory pathway activity in somatic and gametic tissues for a diverse set of 12 vertebrates. We conduct simultaneous gene and TE expression analyses to characterize patterns of TE expression and TE regulation across vertebrates and examine relationships between these features. We find remarkable variation in the expression of genes involved in TE negative regulation across tissues and species, yet consistently high expression in germline tissues, particularly in testes. Most vertebrates show comparably high levels of TE regulatory pathway activity across gonadal tissues except for mammals, where reduced activity of TE regulatory pathways in ovarian tissues may be the result of lower relative germ cell densities. We also find that all vertebrate lineages examined exhibit remarkably high levels of TE-derived transcripts in somatic and gametic tissues, with recently active TE families showing higher expression in gametic tissues. Although most TE-derived transcripts originate from inactive ancient TE families (and are likely incapable of transposition), such high levels of TE-derived RNA in the cytoplasm may have secondary, unappreciated biological relevance.

## Introduction

Transposable elements (TEs) represent the largest identifiable fraction of vertebrate genomes (Chalopin et al. 2015) despite the fact that they are fundamentally mutagens that propagate through the insertion of new copies. Though ubiquitous, the composition and abundance of TEs is highly variable across vertebrate genomes (Chalopin et al. 2015; Kapusta et al. 2017; Pasquesi et al. 2018; Platt et al. 2018). This variability is the result of complex processes acting at both the levels of TEs and the host genome, including population demography (Lynch and Conery 2003; Neafsey et al. 2004; Xue et al. 2018), the evolutionary history of TEs that have infected host genomes (Kordis and Gubensek 1998; Gilbert et al. 2012; Pasquesi et al. 2018), and the ability of the host to repress TE mobilization (Aravin et al. 2007). TE insertions may negatively impact the fitness of their host (Boissinot et al. 2006; Lynch and Walsh 2007) and have been shown to disrupt open reading frames and regulatory regions, alter chromosome structure, and promote genomic rearrangements (Callinan and Batzer 2006; Gasior et al. 2006; Sen et al. 2006; Beck et al. 2011; Vogt et al. 2014; Burns 2017). Yet, increasing evidence for the roles of TEs in rewiring regulatory networks and driving evolutionary innovation (Agrawal et al. 1998; Bourque et al. 2008; Lynch et al. 2015; Chuong et al. 2017; Makałowski et al. 2017; Zeng et al. 2018) counterbalances a simplistic view that TEs are exclusively associated with deleterious impacts on host genomes.

Host genomes have evolved multiple mechanisms to negatively regulate TE activity (i.e., reviewed by Goodier [2016]), with the primary mechanism being epigenetic modification to silence TE-containing chromatin (Reik 2007; Slotkin and Martienssen 2007; Jacobs et al. 2014; Sanchez-Luque et al. 2019). Gonadal germ cell development, however, requires genome-wide erasure of methylation patterns in primordial germ cells to establish cell potency (Surani et al. 2007). This leaves transposons temporarily unsuppressed by chromatin silencing and thus capable of generating heritable insertions until chromatin structure is reestablished (Hajkova et al. 2002; Kato et al. 2007; Molaro et al. 2014). Safeguarding of the genome against this TE propagation in the germline is primarily accomplished by the PIWI:piRNA (PIWI interacting RNAs) pathway (Aravin and Tuschl 2005; Lim and Kai 2015), a specific small RNA interference mechanism that limits TE proliferation at both the transcriptional level through de novo methylation of TE loci and the posttranscriptional level by targeting and degrading TE transcripts (Aravin et al. 2007; Siomi et al. 2011; Weick and Miska 2014).

Previous studies of TE expression and regulation have primarily focused on analyses of critical tissues or temporal windows for novel TE insertions, including testes (i.e., Shi et al. 2007; Handel and Schimenti 2010) and early embryonic-stage tissues (Richardson et al. 2017; Feusier et al. 2019; He et al. 2019). Fewer studies have examined the extent of somatic TE activity (Faulkner et al. 2009; Soumillon et al. 2013; Garcia-Perez et al. 2016; Loreto and Pereira 2017; Faulkner and Billon 2018), although there is evidence for biologically relevant levels of TE transposition in certain somatic tissues, such as the brain, and for elevated levels of TE activation in somatic tissues associated with ageing or disease (Callinan and Batzer 2006; De Cecco et al. 2013; Bedrosian et al. 2016; Faulkner and Garcia-Perez 2017; Kreiling et al. 2017). Currently, our understanding of variation in TE expression and TE regulation across somatic and gametic tissues is based primarily on studies of mammal and bird species (Soumillon et al. 2013), and remarkably little is known about how TE expression and TE regulation may vary across the vertebrate tree of life.

Here, we examine patterns of TE expression and regulation in somatic and gametic tissues from 12 species that represent a sampling of all major vertebrate lineages (supplementary file S1, Supplementary Material online). We leverage this sampling to 1) quantify the effects of conserved TE regulatory mechanisms on TE expression levels within and across vertebrate lineages and 2) evaluate whether nonmammalian vertebrate species follow mammalian patterns of TE regulation and expression. Our integrated analyses provide new evidence for the uniqueness of mammalian germline biology compared with that of other vertebrates, highlight many features of TE regulation shared across vertebrate lineages, and raise new questions about the biological significance of broad expression of TE-derived transcripts in somatic and gametic tissues that appears to be ubiquitous across vertebrates.

## Materials and Methods

We used previously published poly-A-selected RNAseq data sets to compare expression levels of TE-derived transcripts and genes involved in the negative regulation of TEs in testes, ovaries, and up to seven somatic tissues (brain, heart, kidney, liver, muscle, spleen, and small intestine) across 12 vertebrate species that included representatives of fish, amphibians, reptiles, and mammals (supplementary file S1, Supplementary Material online). Additionally, we included available purified oocyte cell data sets for five species (supplementary file S1, Supplementary Material online) for comparison to ovary and testis whole-tissue RNAseq. Raw RNAseq data were first filtered for prokaryote and eukaryote rRNA transcripts using *SortMeRNA* v2.1 (Kopylova et al. 2012), and then quality and adapter trimmed in *Trimmomatic* 0.36 (Bolger et al. 2014). Detailed information for each analysis is provided in the supplementary Methods, Supplementary Material online. For each species, reads were mapped using *STAR* v2.7.0f (Dobin et al. 2013) to the latest genome version and annotation .*gff* files available on the NCBI Genome database (Sayers et al. 2019). All genomes used in this study are associated with high-quality repeat element annotations that incorporated intensive species-specific repeat identification (see supplementary Methods, Supplementary Material online). *STAR* was run using default parameters, discarding chimeric transcripts, and allowing for a maximum of 100 mapped reads per locus (as suggested by Jin et al. [2015]).

Gene and TE-derived transcript expression levels were simultaneously estimated using *TEtranscript* (Jin et al. 2015). To assign mapped reads to a genomic locus, *TEtranscript* requires two annotation files that specify gene and repeat element coordinates, respectively. TE index structures were built from *RepeatMasker .out* files (Smit et al. 2013–2015), and gene index structures were built from the same gene annotation files used when running *STAR* (detailed information on the protocol used to build the input *.gtf* files are provided in the supplementary Methods, Supplementary Material online). *TEtranscript* was run using default parameters, the *–multi* multimapper mode flag, and specifying whether transcriptome data were stranded or not. Expression levels of TE-derived reads that originated from recently active TE copies were estimated in a second, separate analysis (we refer to this as the “recent-TE” data set). This second analysis was required to effectively survey recent TEs because *TEtranscript* analyses do not retain locus coordinates, which prevented us from being able to subsample recent TEs directly from the primary inclusive analysis. For this analysis, we provided *TEtranscript* with a filtered .*gtf* annotation file that contains only TE loci with <2% Kimura two-parameter distance from the consensus. For each species, normalization of TE-derived and gene-derived raw read counts across tissues was performed using the *estimateSizeFactors*-*estimateDispersions-counts(normalized=TRUE)* functions in *DESeq2* v1.20 (Love et al. 2014) after removing elements with <10 mapped reads across all samples. We normalized and performed statistical analyses using both the total-TE and the recent-TE data set and compared normalized gene expression values and results between the two (supplementary file S2, Supplementary Material online). Normalized expression values displayed only minimal variation between total-TE and recent-TE data sets, and statistical analysis results were unaffected, thus we only report results based on normalized expression values associated with the recent-TE data set.

To assess the relationships between TE expression levels and TE regulatory pathway gene levels, we compared recent-TE expression levels with five sets of TE regulatory genes: 1) genes participating in the PIWI:piRNA pathway (Carbon et al. 2009; PIWI pathway hereafter), 2) genes involved in the small RNA interference pathway (Carbon et al. 2009; small interfering RNA [siRNA] pathway), 3) genes involved in transcriptional regulation of TEs (e.g., responsible for de novo DNA or histone methylation; Hutchins and Pei 2015), 4) other genes previously identified to negatively impact TE mobilization and/or insertion at the posttranscriptional level (e.g., Apobec; Goodier 2016), and 5) the combined magnitude all TE regulatory genes (which corresponds to all 79 conserved genes belonging the four previous sets; supplementary file S3, Supplementary Material online).

Patterns of within-species variation in expression levels across tissues were assessed by performing principal component analyses (PCAs) on blind variance stabilizing transformed data (Anders and Huber 2010). Because of the heterogeneous nature of our data, between species comparisons were performed using percentages of the transcriptome following normalization of read counts to limit biases due to different methods of tissue processing, library preparation, sequencing technology, and data set quality (Sudmant et al. 2015; Dunn et al. 2018). To calculate percentages of TE regulatory gene expression, only normalized expression counts derived from annotated genes were used to calculate the total (i.e., TE-derived transcripts were excluded). To investigate relationships in expression patterns across vertebrates, we performed phylogenetic independent contrast (PIC) linear regressions, Spearman rank correlation analyses, and PCAs using the *phytools* package in *R* (Revell 2012). Additional methodological details for statistical analyses performed in this study are provided in the supplementary Methods, Supplementary Material online.

## Results

### TE Regulatory Mechanisms Are Active in Somatic and Gametic Tissues across Vertebrate Lineages

**Fig. 1.**
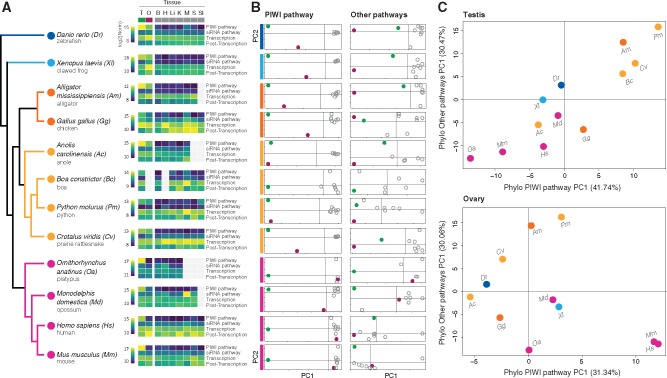
—Expression patterns of key genes involved in negative regulation of TE activity in germline and somatic vertebrate tissues. (*A*) For each species, heatmaps show log 2-transformed within-species normalized expression levels of main pathways involved in TE silencing. Warm colors (yellow) represent higher total expression levels of genes in the pathway across tissues. (*B*) PCAs for the PIWI:piRNA pathway (left) and all other regulatory pathways (siRNA pathway, transcriptional and posttranscriptional TE silencing mechanisms; right) reflect variance in gene expression profiles across tissues for each species. Although nonmammal species show discrimination of both germline tissues (testis in green and ovary in maroon) from somatic tissues (empty gray circles) and from each other in respect to PIWI pathway genes, gene expression in the mammalian ovary falls within the variability of somatic tissues. (*C*) PCAs for the testis (above) and ovary (below) show species clustering based on the principal component of the PIWI pathway (*x* axis) and all other regulatory pathways (*y* axis). Per each species, coordinates were extracted from the corresponding PIC PCAs. Cold colors represent nonamniote vertebrates, warm colors reptiles, and magenta mammal species.

Our analysis of gene expression for a combined set of 79 genes involved in TE negative regulatory mechanisms (supplementary file S3, Supplementary Material online) demonstrates substantial variation in expression across tissues and species. We find that all categories of negative regulators (i.e., repressors of TE activity) are expressed in both somatic and germline tissues at widely varying levels, with roughly 2.5 times higher average expression in the germline (supplementary file S2 and fig. S1, Supplementary Material online and fig. 1*A*). Of all regulatory pathways, the PIWI:piRNA pathway shows higher expression in the germline compared with both somatic tissues (average 16.51-fold higher) and other regulatory gene sets in the germline (1.67-fold higher; supplementary file S2, Supplementary Material online). In contrast, genes involved in the siRNA pathway have consistently low expression in somatic and germline tissues, whereas genes involved in transcriptional and posttranscriptional regulation of TE activity show wide variation in expression across species and tissues (fig. 1*A* and supplementary fig. S1, Supplementary Material online). We also find that negative transcriptional regulators of TE expression on average are expressed at levels similar to the PIWI pathway in the germline, with 2-fold higher expression than in somatic tissues (supplementary file S2, Supplementary Material online). This finding is consistent with elevated levels of chromatin modification and the deposition of histone and DNA methylation markers in germline tissues (Stewart et al. 2016; Greenberg and Bourc’his 2019).

### Patterns of TE Regulatory Mechanism Activation across Tissues and Vertebrate Lineages

To assess variation in expression patterns of TE regulatory pathways among tissues and across lineages, we used multivariate clustering methods to summarize and differentiate trends of expression. Within-species PCAs on gene expression of PIWI pathway genes show distinct, individual clustering of germline tissues in nonmammal species, such that expression patterns in testes and ovaries are distinct from each other and from somatic tissues. In contrast, only testes show a distinct profile in mammals, whereas PIWI pathway levels in mammalian ovarian tissues fall within the variance of somatic tissues (fig. 1*B* left panel and supplementary fig. S2*A*, Supplementary Material online). No clear tissue clustering patterns are observed in pathway-specific analyses of the siRNA, transcriptional, and posttranscriptional regulatory pathways (supplementary fig. S2*B* and *D*, Supplementary Material online), except for a consistent trend of tissue separation driven by the ovary among nonmammal species. Broadly, these other regulatory pathways show cross-tissue profiles similar to those of the PIWI pathway, but with greater variance among somatic tissues (fig. 1*B* right panel). We further measured the contribution of each gene to the principal component determination and find that the five genes with the highest contribution scores all belong to the PIWI pathway for the majority of species (supplementary fig. S3, Supplementary Material online).

To understand how vertebrate lineages differ on the basis of how they regulate TEs in the germline, we directly compared variation in expression levels of TE regulatory pathways between species in germline tissues, specifically. Phylogenetically correct PCAs for the set of PIWI pathway genes, genes from the three other regulatory mechanisms (i.e., “other pathways”), and all mechanisms combined demonstrate distinct TE regulatory mechanism expression patterns in the mammal species analyzed compared with nonmammalian species, largely driven by variation in TE regulatory activity in the ovaries (supplementary fig. S4, Supplementary Material online). Comparisons of the first principal component between the PIWI pathway and “other pathways” distinguish testes gene expression patterns in the alligator and snake species from all other vertebrates (fig. 1*C* above). In contrast, we find that ovary expression patterns in human and mouse cluster independently from other vertebrate species, with the distinction being driven mostly by variation in expression of genes in the PIWI pathway (fig. 1*C* below).

### Between-Lineage Variation in Gametic Tissue Expression of TE Regulatory Pathway Genes

**Fig. 2.**
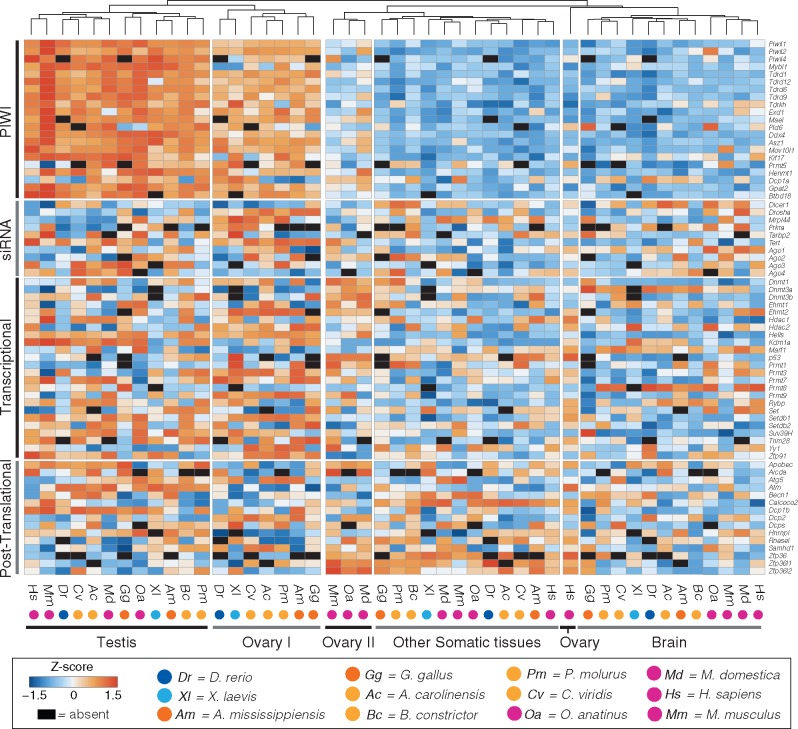
—Hierarchical clustering *Z*-score heatmap of TE regulatory genes in germline and somatic vertebrate tissues. Analysis of differential expression of a subset of key conserved genes (present in at least eight species) involved in TE silencing suggests the existence of five main expression profiles across vertebrate tissues: vertebrate testis, characterized by the highest activation status of the PIWI:piRNA pathway and transcriptional regulators; ovary of nonmammal species, with expression patterns similar to the testis; mammalian ovary (to the exclusion of humans), which shows a sharp decreased expression of PIWI genes; other somatic tissues (average *Z*-scores across heart, kidney, liver, muscle, spleen, and small intestine after individual tissue heatmap supported the existence of a single cluster); and vertebrate brain.

To further characterize variation in TE regulatory activity across lineages, we calculated *Z*-scores of expression relative to the mean expression of all genes for TE regulatory genes with orthologs identified in at least 8 of 12 species (fig. 2). Hierarchical clustering of *Z*-scores across tissues identified five distinct clusters: vertebrate testes, nonmammal ovaries, vertebrate brain, mammalian ovaries, and a mixed cluster of somatic tissues from diverse lineages (fig. 2). In contrast to the single testes germline cluster, we find two clusters of TE regulatory expression profiles from vertebrate ovaries. The first cluster includes all nonmammal species, in which expression profiles resemble TE regulation profiles in the testes. The other cluster is comprised solely of mammals, in which expression levels in the ovary are similar to those observed in somatic tissues. The only exception to this pattern is that the human ovary profile clustered with brain. Differences in relative gene expression levels in vertebrate ovaries are further supported by comparative analyses of differential gene expression between germline and somatic tissues. Multiple genes are significantly differentially expressed in the ovaries of nonmammal species, whereas none is differentially expressed in the mouse or human, and few genes show significant differential expression in the platypus and opossum (supplementary fig. S5, Supplementary Material online).

### TE-Derived Transcript Abundance across Tissues and Vertebrate Lineages

To characterize TE transcription levels and composition across vertebrate tissues, we compared expression levels of total-TE-derived transcripts (total-TE data set; supplementary fig. S6, Supplementary Material online), as well as transcripts derived only from recently inserted TEs (recent-TE data set; supplementary fig. S7, Supplementary Material online). Total-TE expression is substantial in both germline and somatic tissues across all species analyzed, although at variable levels within and between species (supplementary figs. S6 and S8, Supplementary Material online). For example, although total-TE-derived transcripts comprise on average 6.55% of the transcriptome, values range from 0.26% in the chicken muscle to 23.44% in the opossum spleen (supplementary fig. S8 and file S4, Supplementary Material online). Among sampled species, the chicken and human exhibit the lowest total-TE average expression levels (2.66% and 2.92% of the total transcriptome, respectively), due mainly to very low TE transcription levels in somatic tissues (1.52% and 2.35% of the transcriptome on average, respectively). The highest average levels of total-TE expression are found in the two snake species, the prairie rattlesnake and boa constrictor (13.75% and 12.16% of the transcriptome, respectively).

Our analyses also show that germline tissues do not always exhibit higher average total-TE expression levels than somatic tissues in vertebrates. For example, the clawed frog, prairie rattlesnake, platypus, and opossum all exhibit higher average total-TE expression in somatic tissues compared with germline tissues. In the prairie rattlesnake, platypus, and opossum, this is driven by expression levels that are generally elevated in all or several somatic tissues. In the case of the clawed frog, this pattern is driven by comparatively low expression levels of total-TE transcripts in the germline (which are the lowest across all vertebrate species analyzed). Despite high variance in TE expression levels across tissues, several tissues have relatively consistent trends across species. For example, the testes exhibit significantly greater expression on average compared with the ovary (pairwise Wilcoxon test *P* value = 0.02; supplementary fig. S8, Supplementary Material online); this trend is consistent across all species except the opossum, where expression in ovaries is higher than in testes. Additionally, the brain has consistently high total-TE transcription levels across species, which is also higher than expression in testes (average of 9.61% vs. 9.07% of the transcriptomes made up by TE-derived transcripts in the brain and testes, respectively). Conversely, muscle and ovary exhibit consistently low total-TE expression levels (supplementary fig. S8 and file S4, Supplementary Material online). Average expression in the muscle is significantly lower than that of testes (Wilcoxon test *P* value = 0.01), brain (Wilcoxon test *P* value = 0.04), and spleen (Wilcoxon test *P* value = 0.03), and average expression in ovaries is significantly lower than in testes and brain (Wilcoxon test *P* values = 0.02 and 0.04, respectively).

Recent TEs are expressed in both germline and somatic tissues across vertebrates, although at lower levels (0.14% of the transcriptome on average across tissues and species) compared with all TE-derived transcripts (supplementary figs. S8–S10 and file S4, Supplementary Material online). Although lower overall, proportional expression levels of recent TEs are variable across species and tissues (e.g., from 0.003% in boa muscle to 1.94% in zebrafish testes), similar to trends in total-TE transcript levels. However, pairwise comparisons testing for differences in average expression levels across species per tissue were not significant (Kruskal–Wallis rank sum test *P* value = 0.39; pairwise Wilcoxon test *P* values > 0.5). In contrast to the total-TE transcript data set, average recent-TE expression is highest in the testes (0.27%, although this is driven primarily by high testis expression in the zebrafish), followed by the small intestine and the brain (0.22% and 0.19%, respectively). We found multiple examples of divergent levels of recent-TE transcript expression among species within major vertebrate lineages. For example, although mouse exhibits among the highest average recent-TE expression levels, human has low average recent-TE expression levels (supplementary file S4 and figs. S7*C* and S9, Supplementary Material online).

Overall, our analyses demonstrate that recent and total-TE expression levels in somatic tissues are poor predictors of one another. For example, the small intestine has a higher relative fraction of the transcriptome derived from recent TEs, whereas the brain and the spleen have higher fractions of the transcriptome made by TE-derived transcripts that originated from more ancient (and presumably nonmobilizing) TE families (supplementary fig. S9 and file S4, Supplementary Material online). Differences between recent and total-TE expression among germline tissues tend to be clade specific. In the testes, mammal and nonmammal species have similar average total-TE expression levels (8.43% vs. 9.4%, respectively), but remarkably different recent-TE expression levels (0.14% and 0.33%, respectively). With the exception of the zebrafish, however, recent-TE expression levels are very similar (0.14% and 0.10%), in agreement with findings for total-TE expression. In contrast, mammalian ovaries exhibit more than 2-fold greater TE expression than nonmammal species (2.56-fold for recent TEs and 2.16-fold for total TEs; supplementary figs. S9 and S10 and file S4, Supplementary Material online). There is also a positive relationship between the fold-change in TE expression levels (total TE/recent TE) between testes and ovaries at the phylogenetic scale (supplementary fig. S11, Supplementary Material online), and TE-family composition in testis and ovary is very similar for total-TE transcripts. Yet, analyses of recent-TE transcriptional levels highlight sexually dimorphic TE expression, with some specific TE families being expressed exclusively in either ovaries or testes. For example, CR1-LINEs are expressed in the python ovary but not in the testis, and the opposite pattern is observed in the platypus (supplementary fig. S10, Supplementary Material online). Despite tissue-specific expression of some TE families in the recent-TE transcriptome of testes and ovaries, significant associations (linear regression *P* values < 0.005) are still found between relative TE composition of germline tissues for both total and recent-TE expression for each species (supplementary fig. S12, Supplementary Material online).

### Relationships between Genome and Transcriptome TE Composition in Germline Tissues

**Fig. 3.**
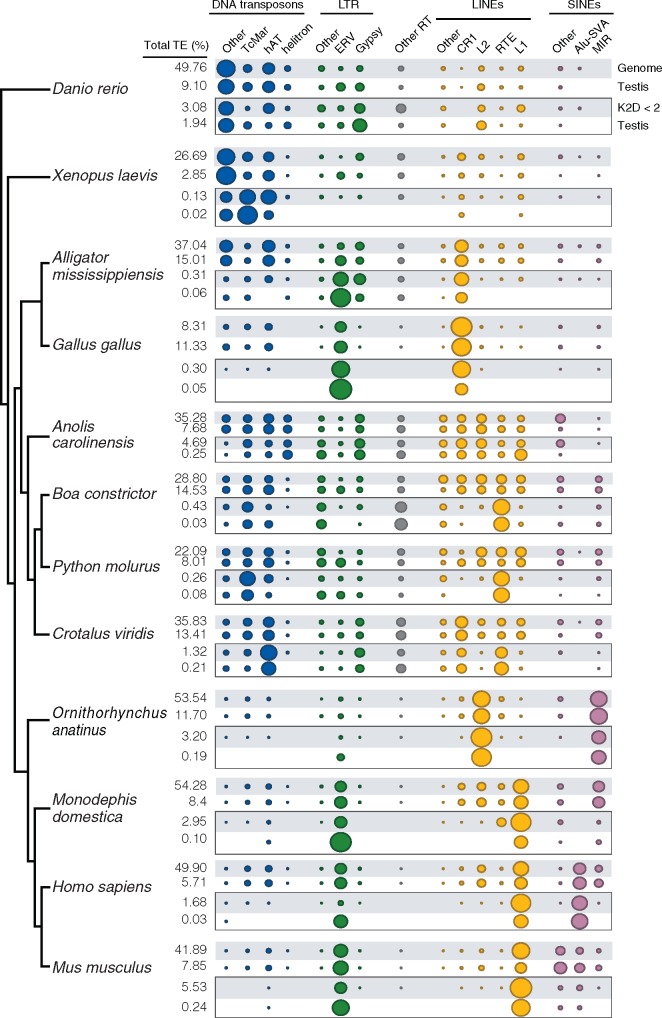
—Relationship between genomic and transcriptomic TE relative composition in the male germline. Area of the circles in the balloon plot reflects the percentage of major TE subfamilies (blue, DNA transposons; green, LTRs; gray, PLE and DIRS; yellow, LINEs; violet, SINEs) relative to the total genomic TE content (top row, gray background) and to the total-TE transcriptome (white background). In the box, the same relationship is displayed for recently inserted TE copies (with a Kimura distance < 2%) and recent TEs in the transcriptome. Values to the left report the real proportion of TEs (TE content %) in the genomes and transcriptomes. We find support for high TE transcription in testis transcriptomes (up to 15%), which perfectly match the relative composition of the genome. In contrast, for recent TEs, some families are entirely missing in the transcriptome despite their presence in the genomic background. Balloon plot additionally highlights variability in TE landscapes across vertebrates.

A stochastic model of genome-wide transcription predicts that a vast majority of the genome is transcribed at some level (Encode Project Consortium 2012; Hangauer et al. 2013). To test whether such a model applies to TEs across vertebrate lineages, we compared relative expression levels of 16 major TE families in the germline with the relative TE composition of the genome for each species analyzed (supplementary file S5, Supplementary Material online). Each vertebrate species is characterized by a strong significant linear relationship between gametic tissue total-TE expression and the relative genomic abundance of TEs for each respective genome (linear regression *P* values < 0.04; supplementary file S6 and fig. S13, Supplementary Material online and fig. 3). We observe similar trends in relative recent-TE transcriptome composition and relative abundance of recently inserted TE copies in the genome (*P* values for all linear regressions are reported in supplementary file S6, Supplementary Material online). However, coefficients of determination are generally lower for recent TEs than for total TEs, and in some species we find a lack of support for the relationship between genome TE content and TE transcriptional levels in the recent-TE-matched comparisons (e.g., chicken, anole, and mouse ovary). This pattern likely stems from multiple instances of TE subfamilies being entirely absent in germline transcriptomes but detectable in the genomes of these species, a trend that is observed in mammals and birds in particular (fig. 3). Finally, comparisons of the relative total genomic TE composition to the relative abundance of recent TEs in germline transcriptomes reveal a lack of associations in testes and ovaries for most species. Mammal species represent an exception to this general trend, however, as they do exhibit significant linear correlations between genomic TE composition and recent-TE expression in both tissues, although with low coefficients of determination (linear regression *P* values < 0.04, supplementary file S6, Supplementary Material online).

### Relationships between Recent-TE Expression and TE Regulatory Activity

**Fig. 4.**
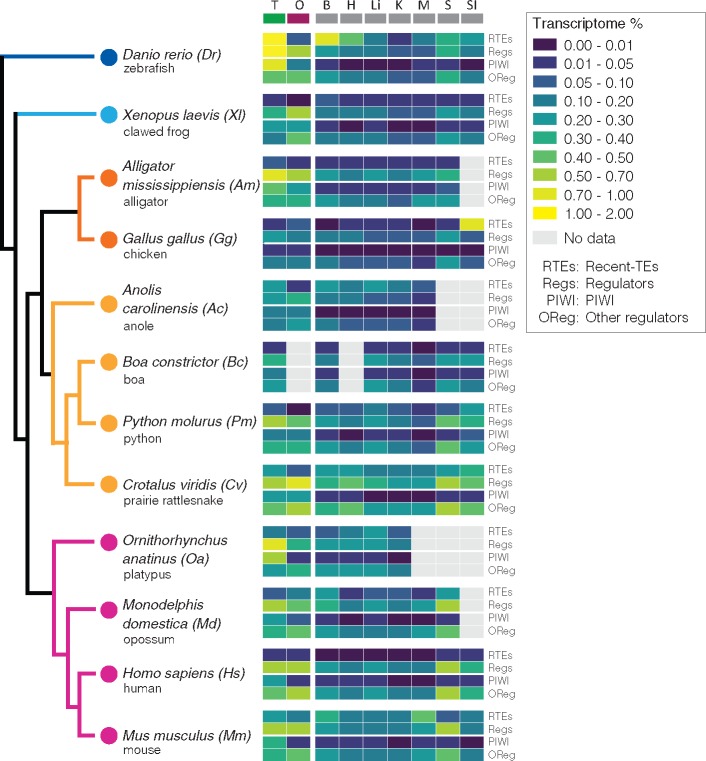
—Expression levels of recent TEs and their negative regulatory mechanisms in vertebrate somatic and germline tissues. Heatmap shows comparative expression levels of recent TEs (top row), total regulatory pathways (PIWI:piRNA, siRNA, transcriptional and posttranscriptional), and details of the contribution of PIWI:piRNA pathway and all remaining silencing mechanism (bottom section) across vertebrate tissues. Comparative gene expression is reported as percentage of the transcriptome following within-species normalization. Whereas human, xenopus, and chicken show the lowest levels of recent-TE expression in both germline and somatic tissues, vertebrate tissues show moderate to high contribution of TEs to tissue transcriptomes, which is consistently highest in the testis, and reduced in nonmammal ovary.

**Fig. 5.**
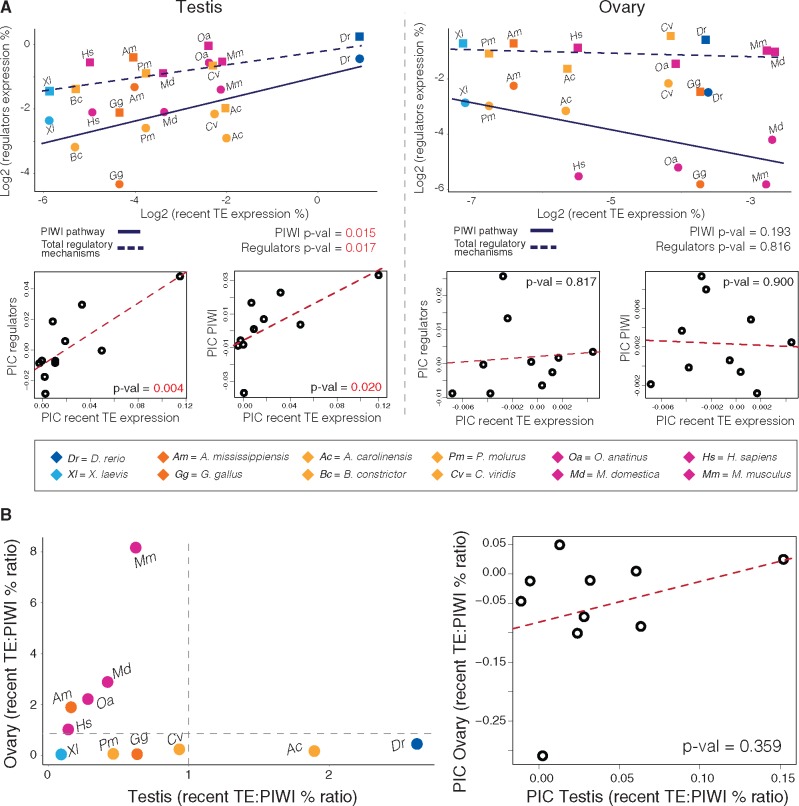
—Relationship between expression levels of recent TEs and their negative regulatory pathways. (*A*) Linear regressions and PICs support a significant positive relationship between recent-TE expression and host response (PIWI pathway and total response) in the testis, whereas in the ovary they suggest the opposite, although not significant, trend. (*B*) Patterns of species TE expression levels in the testis (*x* axis) and ovary (*y* axis). Recent-TE transcriptome percentages were corrected by the PIWI pathway to test for a correlation in expression levels. Mammal species show a consistent trend in the ovary where lower regulatory activity brings to increased TE transcription, matched by the testis although in favor of the PIWI pathway, compared with nonmammal species. In contrast, nonmammal species show a consistent host response proportional to TE activity in the ovary (constant TE:PIWI ratio), but higher variability in the testis, with some species that are more efficient at contrasting TEs.

Considering multiple lines of evidence for differential regulation of TE activity in germline tissues, we tested the relationships between the magnitude of host response against TEs (particularly the relative activation of the PIWI pathway) and recent-TE expression in germline and somatic tissues (fig. 4 and supplementary fig. S14*A*, Supplementary Material online). Relationships were tested using PIC Spearman’s rank-order correlation analyses (supplementary fig. S14 and file S8, Supplementary Material online) and, for germline tissues, PIC linear regression analyses (fig. 5 and supplementary file S7, Supplementary Material online). We found evidence for significant associations between expression levels of recent TEs and both PIWI pathway genes and the entire set of genes involved in TE regulation only in the testes (PIC linear regression *P* values = 0.02 and 0.004, respectively; fig. 5 and supplementary file S7, Supplementary Material online). All other correlation analyses lacked significant associations between recent-TE and regulatory mechanism expression levels, including analyses where somatic and germline tissues were combined and analyses of combined germline tissues (supplementary fig. S14 and file S8, Supplementary Material online). Relationships between recent-TE expression and regulatory activity in ovaries were not significant in analyses of all species as well as analyses in which mammals, which exhibited particularly low expression of PIWI pathway genes and above average recent-TE expression, were excluded (*P* values > 0.05; fig. 5 and supplementary file S4 and fig. S14*D*, Supplementary Material online).

### Comparison of Expression Patterns between Oocytes, Ovaries, and Testes

Our analyses demonstrate broad differences in expression profiles of TE regulatory mechanisms and TE-derived transcripts between testes and ovaries, as well as between mammal and nonmammal ovarian tissues. To evaluate the possibility that our findings are linked to lower fractions of germ cells in ovaries relative to testes, we analyzed available data from purified oocyte cell populations of two nonamniote (zebrafish and clawed frog) and three amniote (chicken, human, and mouse) species. In the zebrafish and clawed frog, oocyte TE regulation and expression profiles recapitulate observations derived from ovarian tissues (supplementary figs. S15 and S16, Supplementary Material online). Expression of genes involved in TE regulation are similar to that observed in the ovary, although oocyte cells have noticeably higher expression of genes belonging to the siRNA pathway compared with somatic tissues (supplementary figs. S15*A* and S16, Supplementary Material online). Similarly, estimates of both total and recent-TE-derived transcript expression appear similar to those of the ovary (supplementary figs. S6*A* and S7*A*, Supplementary Material online). Oocytes also exhibited a complement of high relative activation of TE regulatory mechanisms with comparatively lower recent-TE expression, consistent with observations in ovaries (supplementary fig. S15*A*, Supplementary Material online). In the chicken, oocyte cells share features of TE regulation with the ovary (supplementary fig. S15, Supplementary Material online), yet expression levels of recent TEs are higher than in both ovary and testis. TE regulatory genes in human and mouse oocytes show remarkably distinct clustering patterns (supplementary fig. S16, Supplementary Material online), but in both species we find support for active regulation of TEs (supplementary fig. 15*A*, Supplementary Material online); this is in contrast with profiles of TE regulation in the ovaries. Specifically, expression levels of genes involved in negative TE regulations are more similar to those of the testis than those of the ovary, particularly for PIWI pathway genes. We also find that the oocyte cell populations show unique profiles of total and recent-TE relative expression compared with ovaries and testes in both species (fig. 3 and supplementary fig. S15*C* and file 5, Supplementary Material online). Our results suggest that human oocytes predominantly express Alu SINEs, whereas the testis and ovary show additional relevant contributions of ERV LTRs and L1 LINEs to the transcriptome. In mouse oocytes, most of the recent-TE-derived transcriptome appears to originate from ERV LTRs, but in the ovary and testis L1 LINEs are highly represented as well. Collectively, our analyses suggest that ovaries and oocytes are similar in the zebrafish and the clawed frog but show distinct characteristics of regulatory mechanism and TE expression in human and mouse.

## Discussion

### A Vertebrate-Wide Perspective on TE Expression and TE Regulatory Pathway Activity

To date, studies of TE expression have primarily focused on analysis of male germline and embryonic tissues in mammals (e.g., human and mouse) with the goal of understanding mechanisms that regulate TE activity during developmental stages associated with genome-wide DNA demethylation, and therefore critical for the vertical propagation of TEs (Hajkova et al. 2002; Surani et al. 2007; Ernst et al. 2017; Richardson et al. 2017). Our integrated analyses across germline and somatic tissues shed new light on the variation that exists in both TE expression and regulatory mechanisms among vertebrates and highlight major differences between germline patterns in mammals compared with other vertebrate lineages. Our results also raise new questions about the relatively high, yet variable, levels of TE-derived transcripts across somatic and gametic tissues in vertebrates and underscore the poorly understood relationships between TE regulation and TE transcript expression.

### Overlooked Complexity of TE Negative Regulation in the Vertebrate Germline

Despite major differences in evolutionary history and genomic composition of vertebrate TE landscapes, evidence of active TE repression via multiple conserved regulatory pathways appears to be a shared feature of vertebrates’ somatic and gametic tissues. Expression levels of TE repression mechanisms are particularly variable in ovaries across vertebrate lineages, yet appear to be relatively conserved in the testes. Mammals in particular appear to express genes involved in regulating TE expression in the ovary at a low level similar to expression in somatic tissues, which directly contrasts the active regulatory signature observed in the ovary of other vertebrates. Reduced expression levels of TE regulatory genes in mammalian ovaries may explain why polymorphic TE insertions that have developmental origins in the female early embryo and late germline exhibit the highest transmission rates in mice (Richardson et al. 2017).

These findings raise questions regarding the biological basis and selective drivers that underlie reduced ovarian TE regulation in mammals compared with other vertebrate lineages. One potential explanation may involve differences in mitotic rates in mammals. Previous studies of TE activity and repression have focused specifically on the male germline over the female germline due to higher mitotic and meiotic rates during spermatogenesis (Handel and Schimenti 2010), and other previous studies have indicated that TE activity positively correlates with tissue-specific cell mitotic rates (Navarro et al. 2019). To further explore these relationships, we analyzed expression data derived from oocyte cell populations for five vertebrate species to evaluate if differences in the proportion of germ cells in vertebrate ovarian tissues explain the distinct profiles recovered for mammal and nonmammal species. Although the limited taxonomic sampling prevents us from drawing broad conclusions, our results suggest a relationship between expression patterns of TEs and TE regulatory mechanisms in the ovary and the ratio of somatic-to-germ cells in female gametic tissues. In species characterized by the deposition of numerous eggs (e.g., zebrafish and clawed frog), we found profiles of TE regulatory mechanisms and TE expression in oocytes to be remarkably similar to those of the ovary. These findings agree with the existence of an ovarian germline stem cell (OGSCs) population to replenish the oocyte pool (Hanna and Hennebold 2014). In contrast, human and mouse (where the presence of OGSCs is still debated; Hanna and Hennebold 2014) show profiles of TE expression and regulation that more closely resemble testis profiles than those of ovary, suggesting a lower fraction of germ cells and their precursors in the ovary. Although chicken oocyte cells share features of TE regulation with the chicken ovary, our analyses seem to agree with previous findings that do not support an OGSC population (Motono et al. 2008; Nakamura et al. 2013).

Across animals, ovaries are characterized by a cell population in meiotic arrest (Sagata 1996). Our analyses provide indirect support that differences likely exist not only in the frequency and magnitude of oocyte activation across lineages (Abrieu et al. 2001) but also between germ cells at the same maturation stage of closely related species (i.e., MII oocytes of human and mouse). Future study of the variation in key features of ovarian biology across vertebrates, including mitotic and meiotic rates, stage of oocyte maturation at the onset of meiotic arrest, as well as the presence of OGSCs, may prove valuable for examining links between variation in characteristics of ovarian biology and the activity of TE regulatory mechanisms across vertebrate lineages.

Few previous studies have focused on TE regulatory mechanisms outside the mammalian germline (Watanabe et al. 2008; Lim et al. 2013; Malki et al. 2014), which limits the context for comparison of our results across tissues in vertebrates. Our conclusion that PIWI pathway genes are expressed at similar levels in testes and ovaries is broadly consistent with previous studies in the zebrafish, clawed frog, and anole (Houwing et al. 2007; Kirino et al. 2009; Zhang et al. 2017), whereas expression of PIWI mRNAs or piRNAs has not been detected in previous studies of chicken ovaries (Sun et al. 2017). Interestingly, the zebrafish is also known to produce sex-specific piRNAs from distinct genomic TE loci (Zhou et al. 2010); if this mechanism exists in other vertebrates, it may provide an explanation for sexually dimorphic expression of recent TEs in the germline.

### TE Regulatory Pathways Do Not Clearly Demarcate Somatic and Gametic Tissues

Our comparative analyses illustrate that expression of genes involved in the negative regulation of TEs is not limited to the germline. Among the four categories of TE regulatory mechanisms analyzed, only expression levels of the PIWI:piRNA pathway consistently discriminated at least one germline tissue from somatic tissues based on variation in across-tissue gene expression. In contrast, endogenous siRNA, transcriptional, and posttranscriptional pathways are all characterized by relatively consistent expression levels across germline and somatic tissues. Our analyses therefore support the canonical view of PIWI pathway genes and associated piRNAs are a hallmark of gametic tissues, and the vanguard of germline genome integrity.

TE expression and TE repression mechanisms have been extensively studied in somatic tissues, but mostly in association with cancer, aging, and other diseases (Kazazian 1998; Burns 2017; Kreiling et al. 2017; Jang 2019). Those studies have led to the collective view that, because of the threat that TE mobilization poses to genome integrity and structure, their expression is severely restricted at both transcriptional and posttranscriptional levels. Subsequent studies found exceptions to this pattern in the central nervous system and in specific developmental stages, where expression of specific elements promotes cellular mosaicism and the correct execution of cell specification pathways, respectively (Baillie et al. 2011; Weissman and Gage 2016; Hackett et al. 2017). Broadly, our findings indicate that genes traditionally associated with the germline (e.g., genes in the PIWI:piRNA pathway; Ponnusamy et al. 2017) also exhibit detectable expression in somatic tissues, although often at low levels, and vice-versa (e.g., genes in the siRNA pathway; Stein et al. 2015).

The brain is the only somatic tissue where de novo TE insertions have been identified outside the germline in nonpathological conditions (e.g., Baillie et al. 2011; Weissman and Gage 2016) Understanding how TE activity is regulated in the central nervous system is therefore a topic of primary interest. In the context of our study, we find a single, distinct profile of TE regulation common to all vertebrate brain tissues characterized by higher relative expression of transcriptional regulators (e.g., TRIM28 and methyltransferases). This finding suggests that a conserved landscape of TE activity may exist in the central nervous system across vertebrates. PIWI genes and most members of the PIWI:piRNA pathway show little to no expression in the brain, suggesting that this regulatory mechanism does not play a role in TE regulation in the brain across vertebrates (supplementary figs. S1 and S17, Supplementary Material online), and that other repressive mechanisms may have evolved to regulate TE mobilization in the central nervous system (Grassi et al. 2019). These results further support the roles of TE regulation in somatic tissues, possibly through the evolution of compensatory or reinforcing mechanisms, or the cooption of existing mechanisms for TE regulation (Levine et al. 2016).

### Interpretations of TE-Derived Transcript Abundance

Our analyses demonstrate that TE-derived transcripts on average comprise a notably large fraction of the transcriptomes of germline and somatic tissues across vertebrate lineages. We expected a priori that a majority of TE-derived transcripts would originate from recent active TE families, yet this pattern was not observed in any of the species analyzed. Instead, TE-derived transcripts originate from a variety of recent and ancient TEs families among the species studied. These findings, corroborated by the identification of similar relative composition of genomes and TE transcriptomes across species, support hypotheses from studies in mammals evoking a stochastic transcription model, in which the majority of the genome is pervasively transcribed (Encode Project Consortium 2012; Hangauer et al. 2013). Although the majority of TE-derived transcripts may not have biological activity related to insertional mutagenesis or replication, it remains an open question whether the abundant pool of TE-derived cellular RNAs have other biologically relevant impacts in gene regulation (e.g., lnRNAs and microRNAs), unappreciated roles due to their sheer abundance (e.g., mass-effect competition for RNA catabolic processes, RNA metabolism, and interference with translation) or potential cooption as regulatory elements (van de Lagemaat et al. 2003; Lippman et al. 2004; Cordaux and Batzer 2009; Chuong et al. 2017). It is notable that particular somatic tissues in some species exhibit distinctly high estimates of TE transcripts (e.g., 23.44% in opossum spleen). Because our approach cannot differentiate between legitimate TE-mRNAs and transcriptional read-through, it is unclear whether one or both of these may explain these high expression levels. Specifically in the case of spleen tissues, it is plausible that our inferences of TE-derived transcripts may be confounded by TE-related gene expression (e.g., recombinase activating genes) in tissues with high levels of immune cell activity.

To focus on TE-derived transcripts that are likely to be relevant sources of mutation and transpositional activity, we restricted our analyses to transcripts that originated only from recently active TEs (i.e., recent TEs). Such recent TEs are likely to be more strongly targeted by negative regulatory mechanisms (Vandewege et al. 2016; Sun et al. 2017). We find that recent TEs are expressed in both germline and somatic tissues across vertebrates, although at far lower levels (mean = 0.14% of the transcriptome) compared with total-TE-derived transcripts (mean = 6.55%). Recent-TE expression tends to be highest in the testes, followed by the small intestine and the brain. Our results also indicate that patterns of recent-TE expression in mammals are unique among vertebrates analyzed. Mammals have relatively higher levels of expression in the ovaries, such that mammalian ovaries and testes show similar recent-TE expression levels. We also identified multiple examples of highly divergent levels of recent-TE transcript expression among species within major lineages, suggesting that substantial variation likely exists across species within major vertebrate lineages.

Variation in genome assembly quality may impact reconstructions of genomic TEs, especially recent TEs. Although this is expected to impact estimates of genomic TE content for recent TEs, this should have a minor impact on inferences of TE activity as long as at least some copies of recent-TE sequences are reconstructed correctly for each repeat type per genome. Accordingly, our genome-wide mapping of RNAseq reads to annotated genomic repeat sequences to infer transcript levels for each type of TE should be robust across genome assemblies of reasonable quality, because our inferences of transcriptional activity of TE types are not dependent on the number of recent-TE copies, but rather on having at least some closely related examples of each TE type present in the genome annotation.

Our analyses of the relative composition of TE-derived transcripts in vertebrate gametic tissue transcriptomes highlight extreme variability in evolutionary TE dynamics across lineages. Some species (e.g., squamate reptiles) show fairly equal representation of all major TE subfamilies in their recent-TE-derived transcriptomes. Other species, including human, mouse, and chicken, have been characterized by the extinction of most TE families, such that few elements are thought to remain capable of generating novel insertions (Gagnier et al. 2019). In human and mouse, we inferred traces of DNA transposon-derived transcripts in the recent-TE data set, which we expect likely represent results derived from transcriptional read-through. On a broad level, however, our analyses agree with recent studies (e.g., Feusier et al. 2019) suggesting high retrotransposition rates in the human germline of L1 LINEs and Alu/SVA SINEs. We also found that human testes, ovaries, and oocytes have different relative recent-TE-derived transcripts composition, with L1 and Alu/SVA transcripts being present in the testis (30% and 41% of the recent-TE transcriptome, respectively), yet in oocytes we estimated Alu/SVA transcripts as comprising up 92% of the recent-TE transcriptome. Similar sexual dimorphism in relative recent-TE-derived transcript composition was also observed in mouse and chicken, but not in zebrafish or clawed frog. Substantial differences therefore exist in TE expression and TE regulation between male and female germline tissues, and relationships between sex-specific germline tissue expression patterns further vary across vertebrate lineages.

Collectively, our analyses of the relationships between recent-TE expression and TE regulatory pathway activity provide evidence for divergent patterns between gametic tissues across vertebrates. In the male germline, there is a positive relationship across vertebrates between expression levels of recent TEs and TE regulatory pathway activity. Given how the PIWI:piRNA pathway acts to repress TE (i.e., Lim and Kai 2015), these findings may suggest that activation of TE repressive mechanisms may be proportional to the magnitude of threat posed by TE expression and activity. This is consistent with previous findings that higher TE activity is associated with higher TE repressive mechanism activation in the host (Reznik et al. 2019). In contrast, no significant relationship was found between the expression of recent TEs and TE repressive mechanisms in vertebrate ovarian tissues, suggesting that the unique biology of the ovary may confer or necessitate unique mechanisms to prevent the potentially deleterious effects of TE activity.

### Conclusions and Future Directions

Our comparative analyses of TE regulation and expression across vertebrate lineages suggest that active repression of TEs is accomplished by multiple conserved mechanisms and represents a shared feature among germline and somatic vertebrate tissues. Our results also highlight highly unique sexually dimorphic TE-associated biology specific to gametic tissues. We find that patterns of TE regulation are remarkably distinct in mammalian ovarian tissues compared with other vertebrates, and that a shift toward decreased TE regulatory activity in ovaries occurred early in the evolution of the eutherian mammal lineage. Yet, analyses of oocyte cells for two mammal species suggest the possibility that lower expression levels of TE regulatory mechanisms may be due to a lower proportion of germ cells in the eutherian mammal ovary compared with other vertebrate species. These findings, together with other differences in TE regulation and expression identified among vertebrate lineages, underscore the importance of studies of diverse vertebrate lineages and tissues for understanding the uniqueness of mammalian biology and demonstrate the potential shortcomings of broad assumptions that diverse vertebrate model systems share common biological features and regulatory mechanisms. Our findings also underscore challenges in understanding the relevance of TE-derived transcript abundance from analysis of RNAseq data alone, argue for future integration of approaches that quantify transpositionally competent TE-derived transcripts (Deininger et al. 2017), allow for a better discrimination of TE-loci transcription and gene read-through, and leverage other functional data (Faulkner et al. 2009; Sun et al. 2017; He et al. 2019).

Although our analyses focused on TE negative regulatory pathways and how they relate to expression levels of recent-TE-derived transcripts, instances of genes and transcription factors that can promote TE activity (e.g., Runx3; Yang et al. 2003), or both negatively and positively regulate TEs in a context-specific fashion (e.g., YY1; Athanikar et al. 2004; Sanchez-Luque et al. 2019) have been reported. While beyond the scope of our current study, we cannot dismiss that different expression levels of genes that promote TE transcription may result in difference in expression levels of specific TE subfamilies across tissues (e.g., supplementary fig. S18, Supplementary Material online). Investigation of such positive TE regulation mechanisms would be a valuable complement to our analyses of negative TE regulation.

## Supplementary Material

evaa068_Supplementary_Data

## Notes

**Data deposition:** New unassembled RNA sequencing data have been deposited at NCBI under the accession PRJNA608231. The authors declare that all data and scripts used in this study are available via public databases or available from the corresponding author upon request.
