# Supplementary material for: Vertebrate Lineages Exhibit Diverse Patterns of Transposable Element Regulation and Expression across Tissues
Source: Genome Biol Evol. 2020 Apr 9;12(5):506–21. doi: 10.1093/gbe/evaa068 (PMC7211425; doi:10.1093/gbe/evaa068)
Supplement: evaa068_Supplementary_Data [file evaa068_supplementary_data.zip › SupplementaryFig_03.02.2020.pdf]

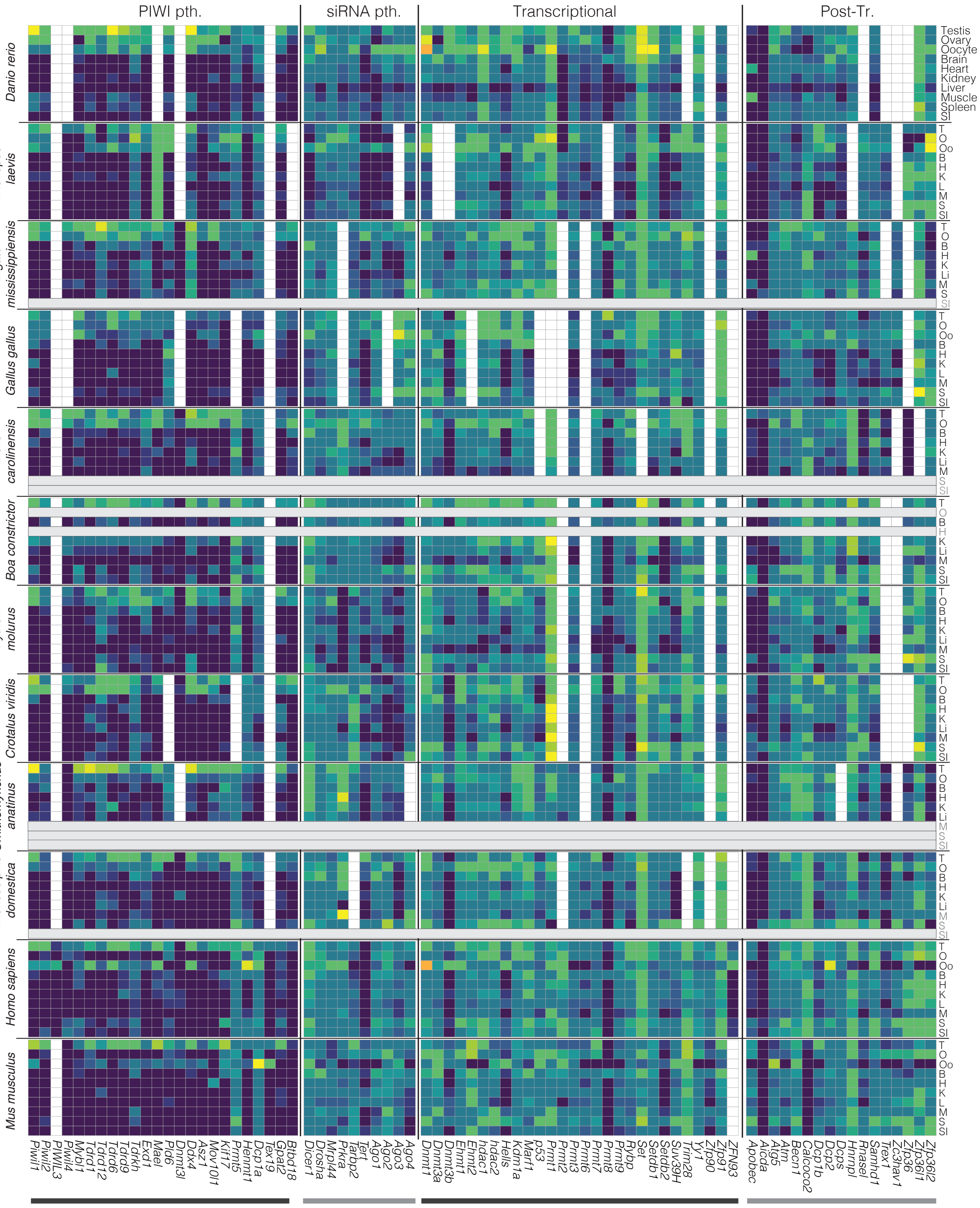

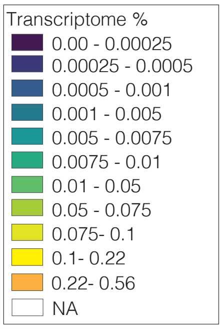

Supplementary fig. S1. Estimates of negative transposable element (TE) regulators expression levels in somatic, germline tissues, and oocytes across vertebrate species. Heatmap shows variation in expression levels of genes belonging to the PIWI:piRNA pathway (PIWI path.), small interfering RNA (siRNA path.), negative transcriptional and post-transcriptional (Post-Tr.) regulation of TEs. We used percentages of within-species normalized gene expression levels as estimates of gene expression to account for between species library variation.

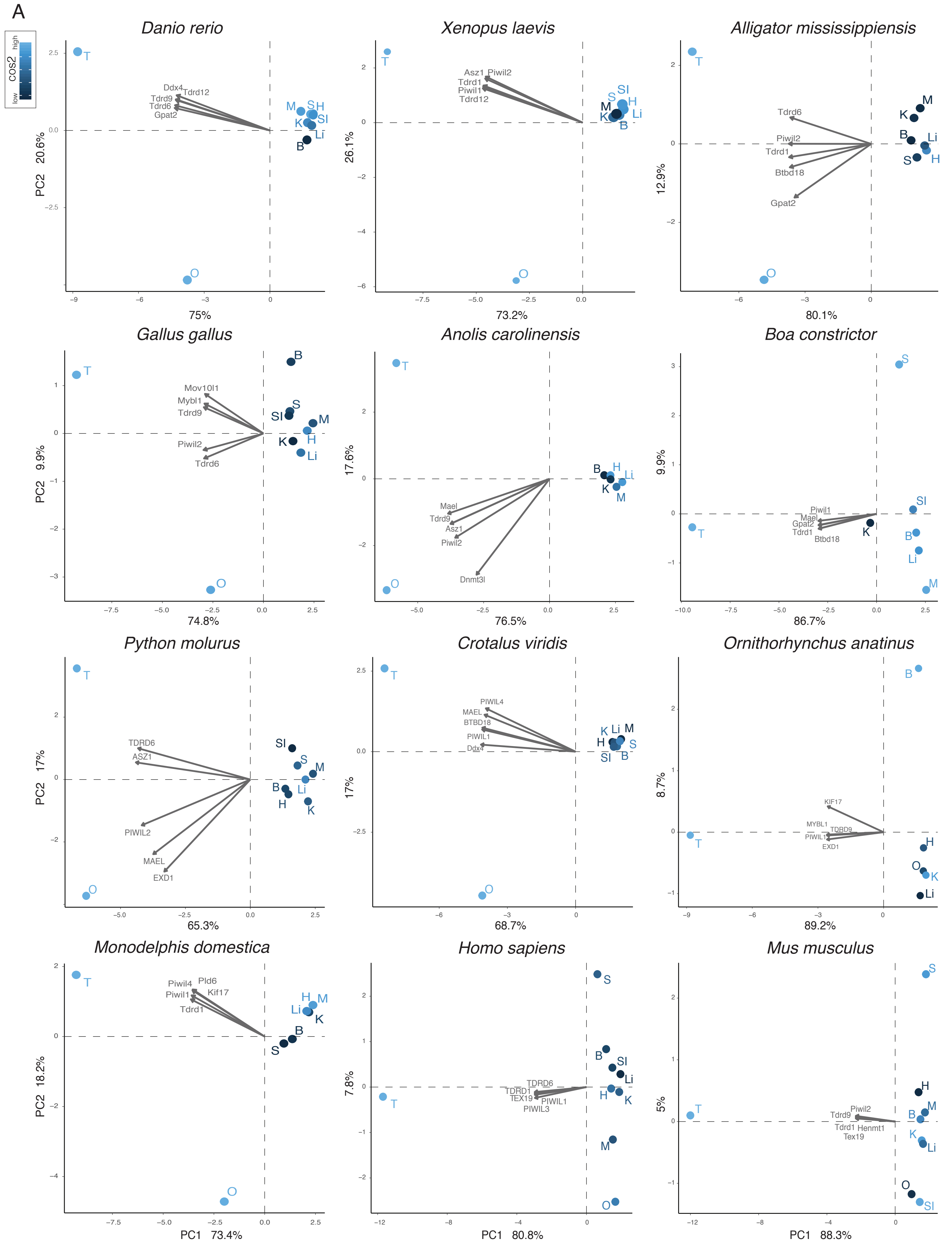

Supplementary fig. S2A. Principal Component Analyses (PCA) for genes taking part in the PIWI-piRNA pathway of negative TE regulation across tissues in vertebrate species. PCA plots show tissue clustering based on variance stabilized transformation (vst) of gene expression estimates assessed in DeSeq2. Tissues are colored according to their contributions (cos2 = quality of the sample on the factor map). Arrows represent the eigen vectors for the 5 most contributing variables in the variables factor map. Noticeable is the discrimination of both germline tissues in non-mammal species, whereas only the testis is discriminated in mammals.

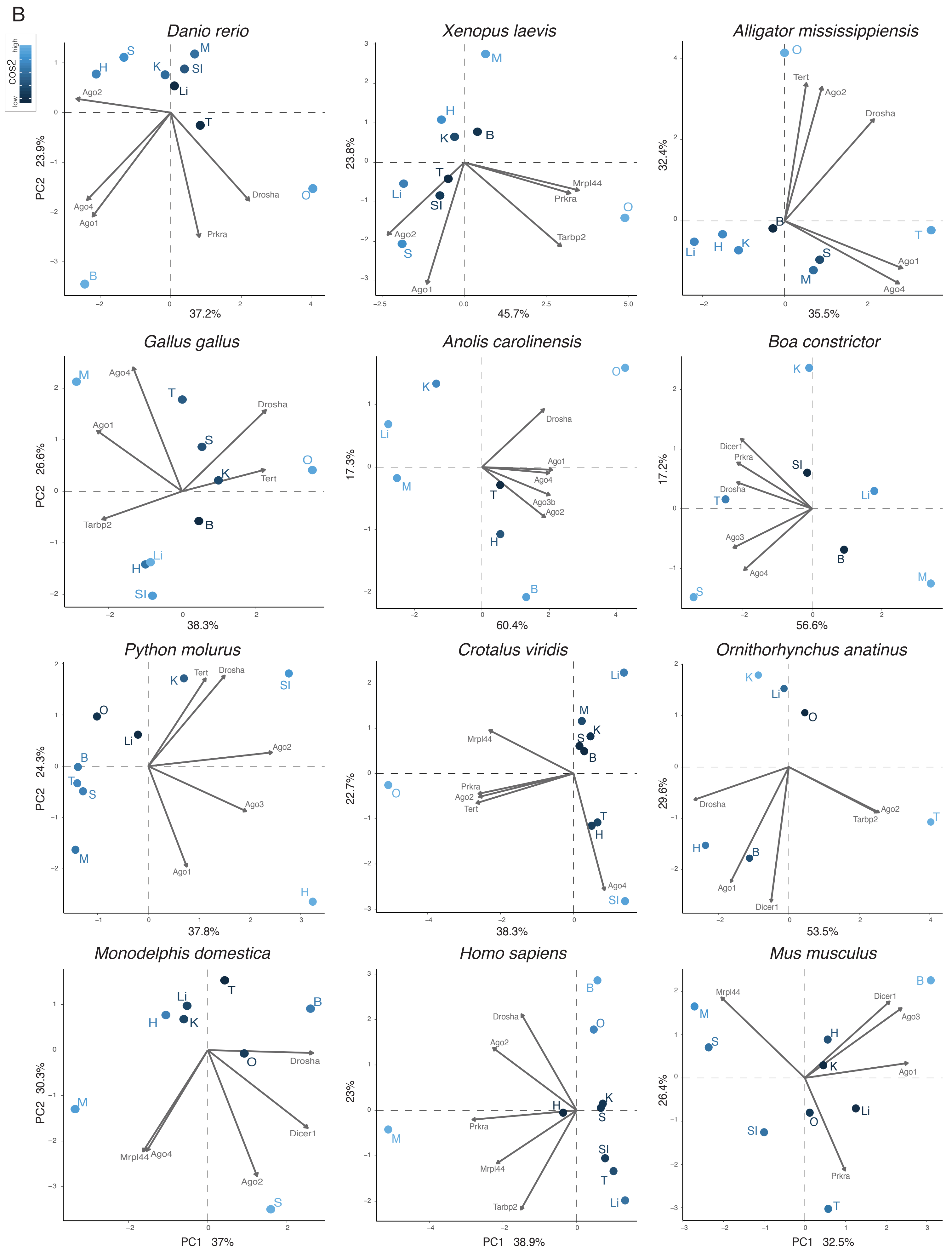

Supplementary fig. S2B. Principal Component Analyses (PCA) for genes taking part in the small interfering RNA (siRNA) pathway across tissues in vertebrate species. PCA plots show tissue clustering based on variance stabilized transformation (vst) of gene expression estimates assessed in DeSeq2. Tissues are colored according to their contri-butions (cos2 = quality of the sample on the factor map). Arrows represent the eigen vectors for the 5 most contribut-ing variables in the variables factor map. In contrast to PIWI pathway gene PCAs, variance in gene expression levels between somatic tissues is greater than between germline and somatic tissues.

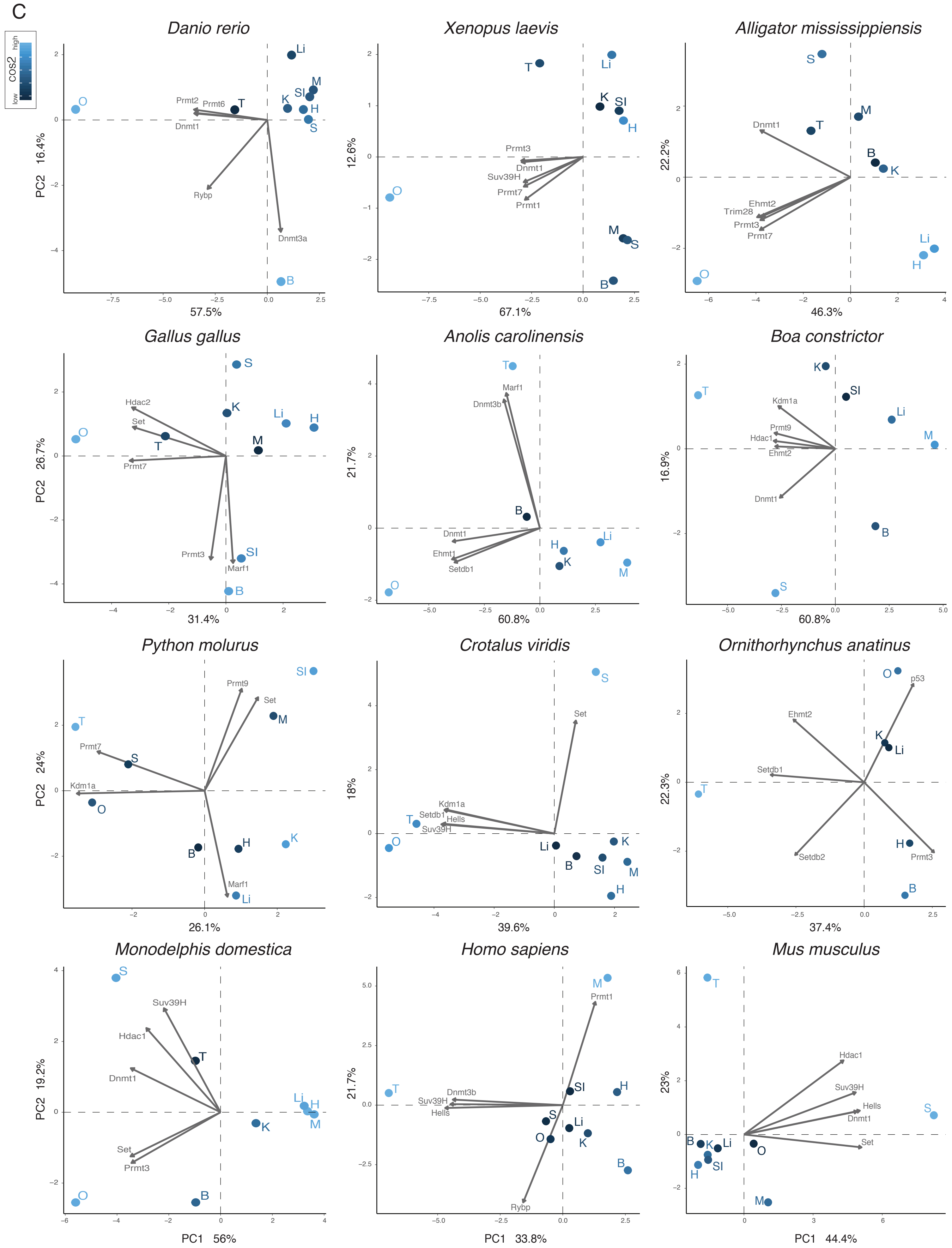

Supplementary fig. S2C. Principal Component Analyses (PCA) for genes taking part in negative transcriptional regulation across tissues in vertebrate species. PCA plots show tissue clustering based on variance stabilized transformation (vst) of gene expression estimates assessed in DeSeq2. Tissues are colored according to their contributions (cos2 = quality of the sample on the factor map). Arrows represent the eigen vectors for the 5 most contributing variables in the variables factor map. Whereas variance in gene expression levels between somatic tissues varies between species, the ovary is characterized by the highest variance in most vertebrates to the exception of eutherian mammals.

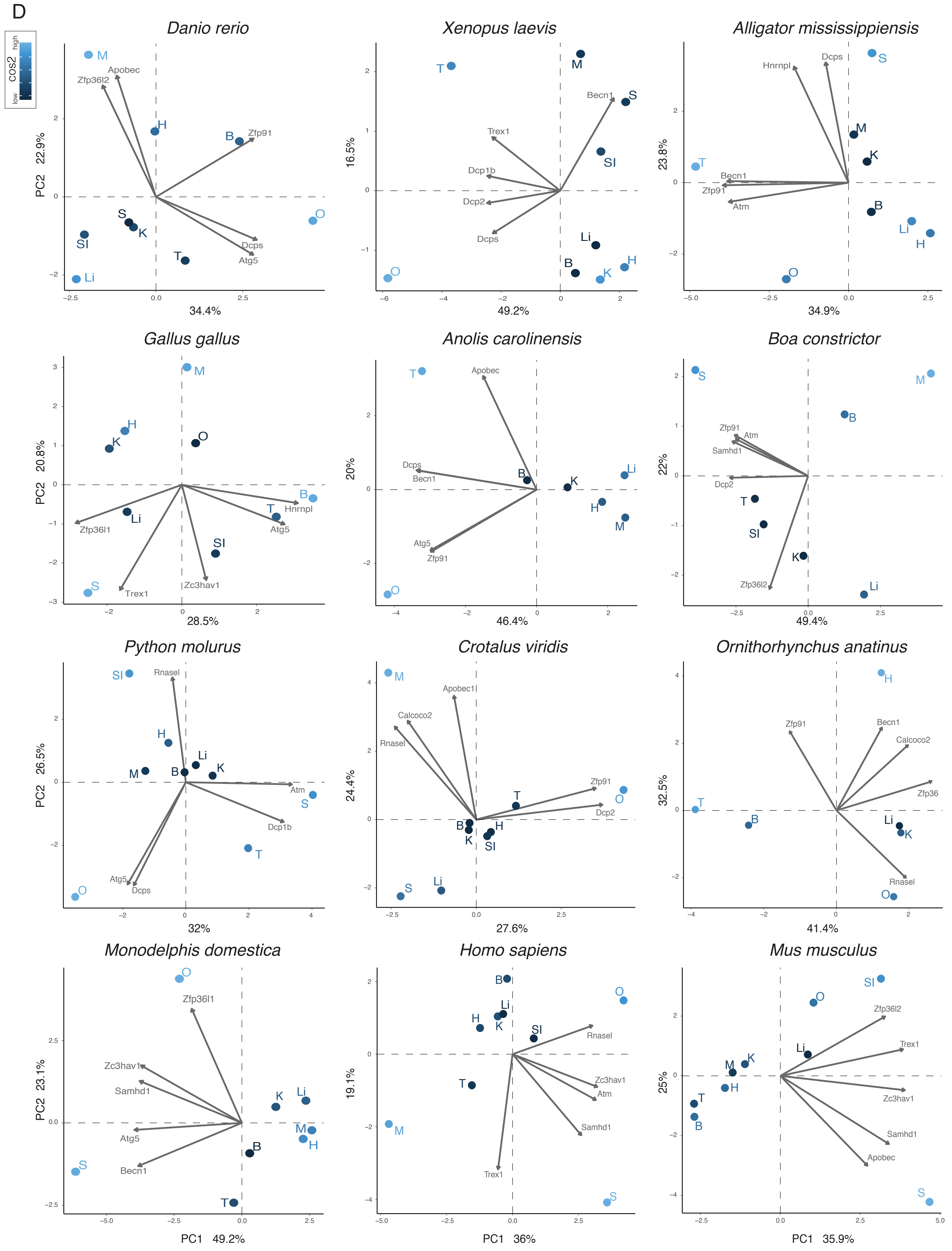

Supplementary fig. S2D. Principal Component Analyses (PCA) for genes taking part in negative post-transcriptional regulation across tissues in vertebrate species. PCA plots show tissue clustering based on variance stabi-lized transformation (vst) of gene expression estimates assessed in DeSeq2. Tissues are colored according to their contributions (cos2 = quality of the sample on the factor map). Arrows represent the eigen vectors for the 5 most contributing variables in the variables factor map.

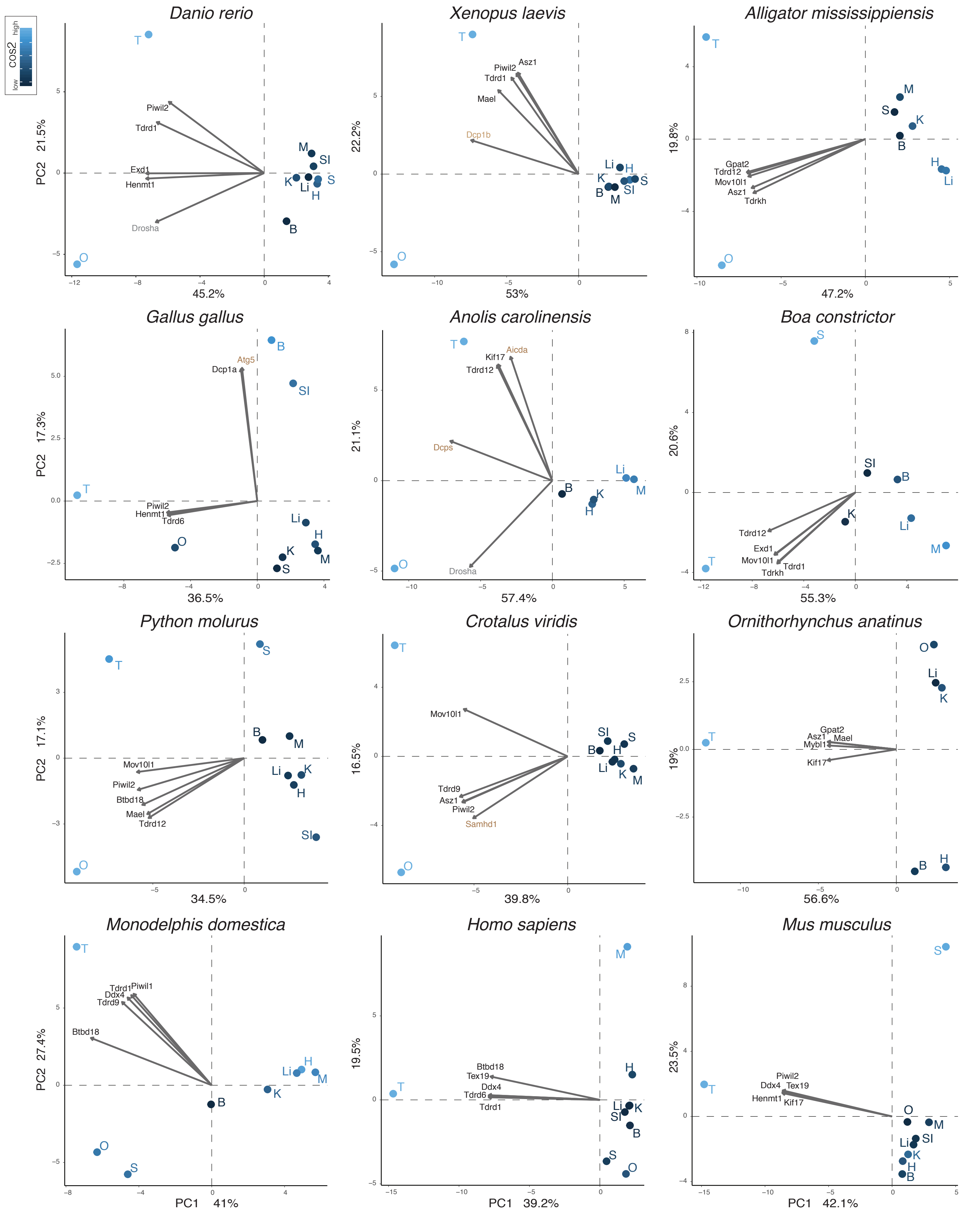

Supplementary fig. S3. Principal Component Analyses (PCA) for genes taking part in negative TE regulation across tissues in vertebrate species. PCA plots show tissue clustering based on variance stabilized transformation (vst) of gene expression estimates assessed in DeSeq2. Tissues are colored according to their contributions (cos2 = quality of the sample on the factor map). Arrows represent the eigen vectors for the 5 most contributing variables in the variables factor map. The evolutionary transition to mammals is mirrored by a decrease in variance and contribution of the ovary compared to other somatic tissues, whereas in non mammal species both germline tissues cluster independently with high support. Genes belonging to the PIWI:piRNA pathway (black) appear to be major factors driving tissue separation relatively to genes belonging to the siRNA pathway (gray), transcriptional (brown), and post-transcriptional mechanisms (light brown).

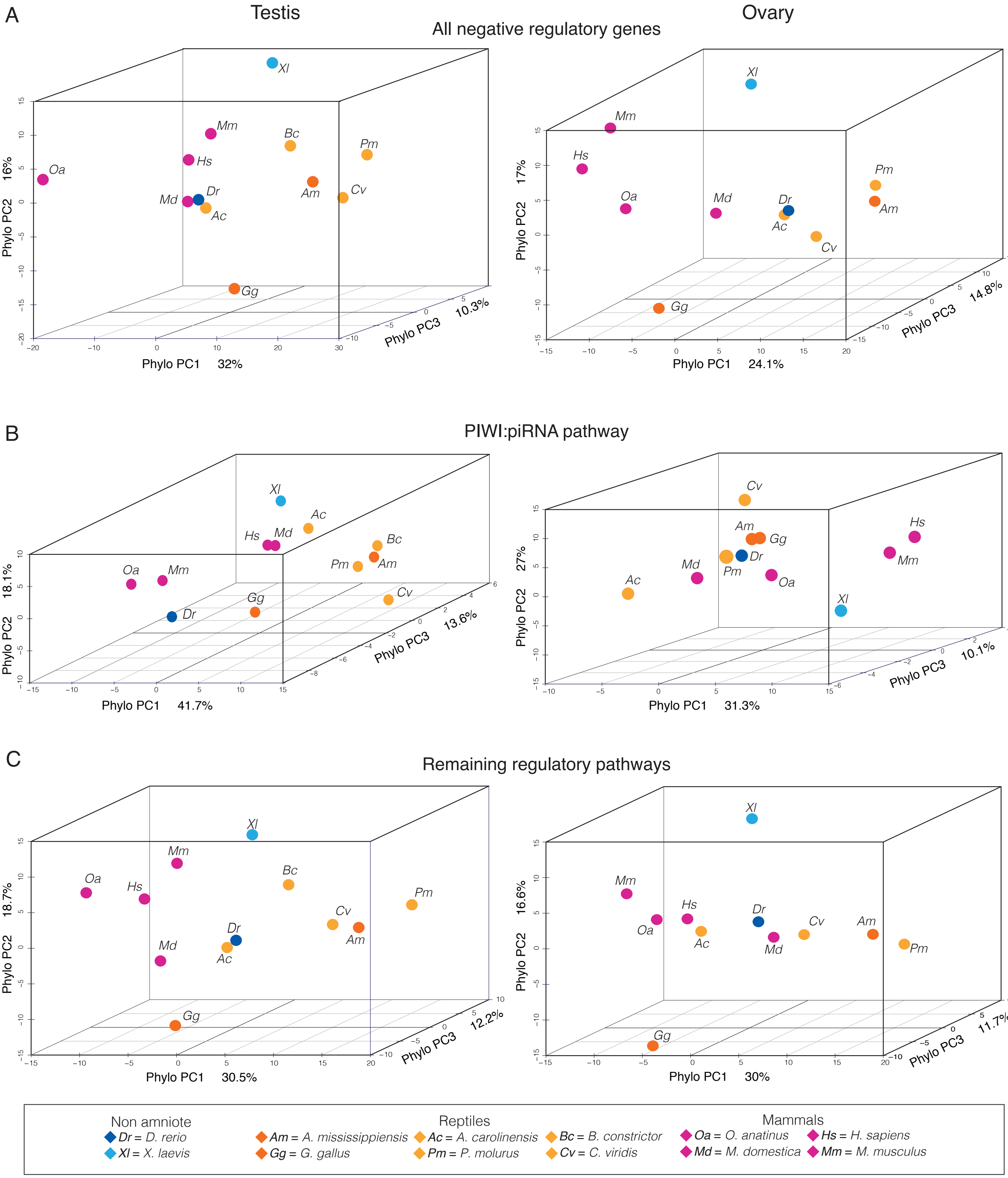

Supplementary fig. S4. Pylogenetic PCAs for expression levels of genes involved in negative regulation of TEs in germline tissues. A-C) Phylogenetic PCAs show clustering of vertebrate species according to variance in vst (variance-stabilized transformation) gene expression levels in the testis (left) and in the ovary (right). A) When all genes are considered, mammals (eutheria in particular) form a distinct cluster for the ovary, whereas clustering structure is less pronounced in the testis. A similar trend is observed for the genes involved in the PIWI:piRNA pathway (B), but exclusively for eutheria mammals in the ovary. C) Remaining regulatory pathways include genes that take part in the siRNA pathway and in transcriptional and post-transcriptional regulation of TEs. Clustering of mammals is present in both germline tissues, but more evident in the male than in the female germline. Overall, squamates tend to cluster together in all PCAs, except for the chicken that, like the clawed frog, tends to cluster individually in A and C. Results support our hypothesis of differential regulation of TE expression in germline tissues between mammals and other vertebrate species.

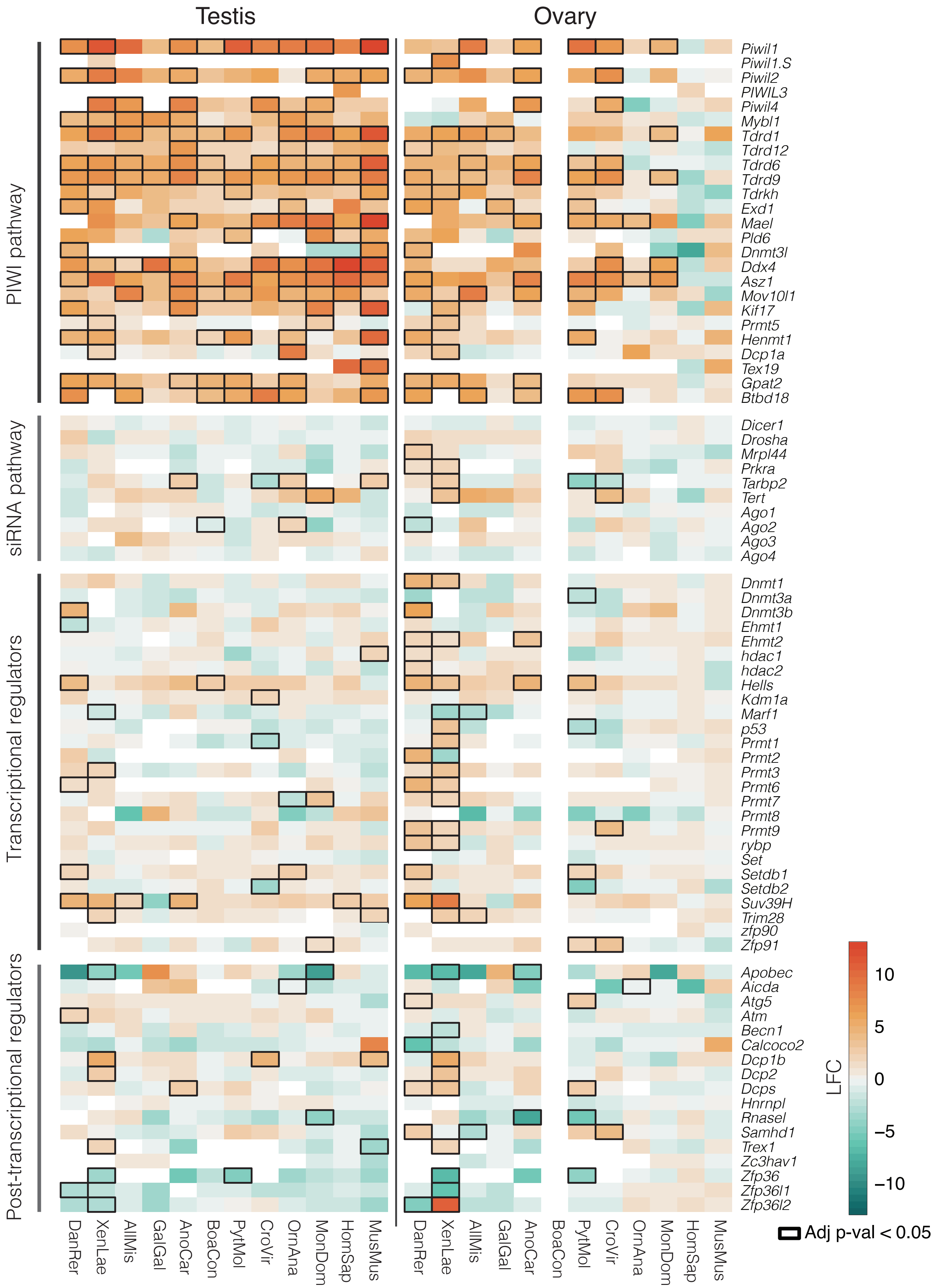

Supplementary fig. S5. Germline differential expression of genes involved in negative TE regulative pathways. Heatmap shows log fold changes (LFC) in expression levels of main negative TE regulators in germline tissues (testis on the left and ovary on the right) compared to their somatic expression. Warmer colors represent enrichment in the germline, and colder colors represent lower expression. Significant differential expression (DE) is reported as black cell outlines. DE analyses were performed in DEseq2. In agreement with the literature, we detected a significant enrichment for genes involved in the PIWI:piRNA pathway in the male germline across all vertebrate species, but mainly across non mammal species in the female germline.

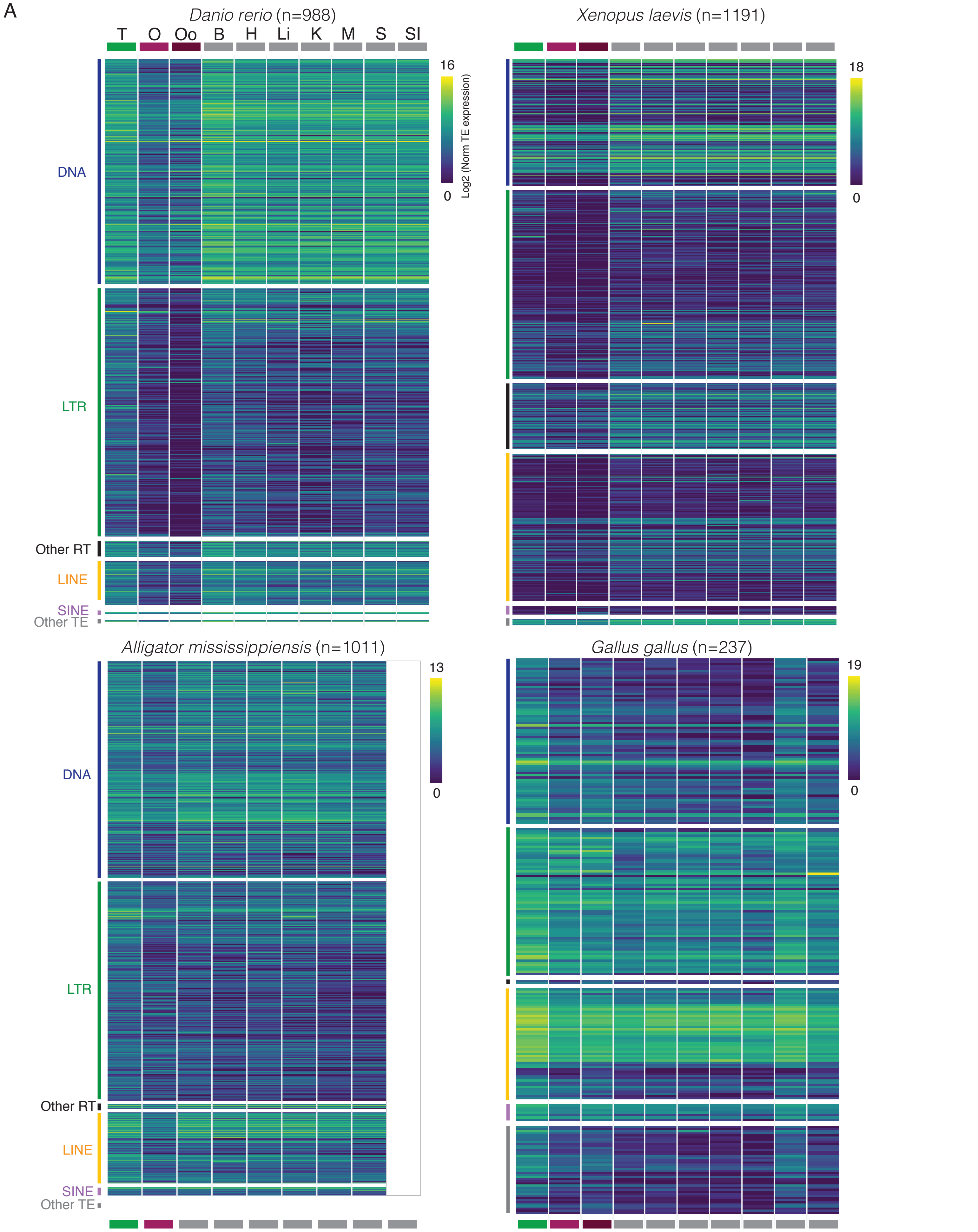

Supplementary fig. S6A. Total-TE derived transcript expression across tissues. Heatmaps show individual TE expression levels across tissues for lower vertebrates and archosauria reptiles. Empty columns represent tissues with unavailable transcriptome data. Hearmaps also reflect the relative abundance in terms of number of individual elements belonging to each of the major TE classes. T = testis; O = ovary; Oo= oocyte; B = brain; H = heart; Li = liver; K = kidney; M = muscle; S = spleen; SI = small intestine. LTR = Long Terminal Repeats; Other RT = other retrotransposons (PLE and DIRS); LINE = Long Interspersed Nuclear Elements; SINE = Short Interspersed Nuclear Elements. To the exception of the chicken, TE expression levels are consistent across tissues for the other vertebrate species.

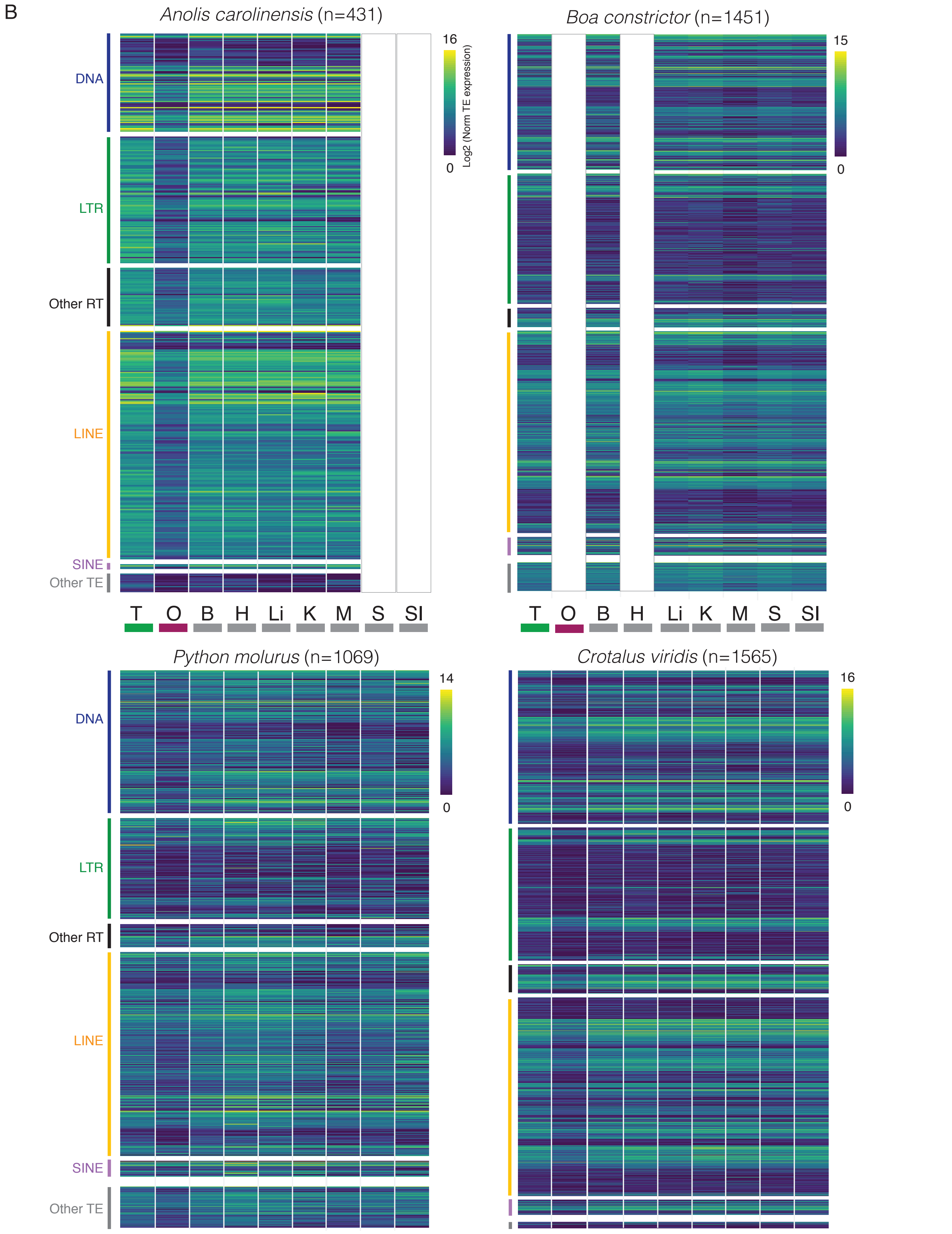

Supplementary fig. S6B. Total-TE derived transcript expression across tissues. Heatmaps show little variability in individual TE expression levels across tissues in squamate reptile species, although some tissues like the ovary in the green anole lizard (*Anolis carolinensis*) and in the prairie rattlesnake (*Crotalus viridis*), and the muscle in *Boa constrictor* are characterized by overall lower expression levels.

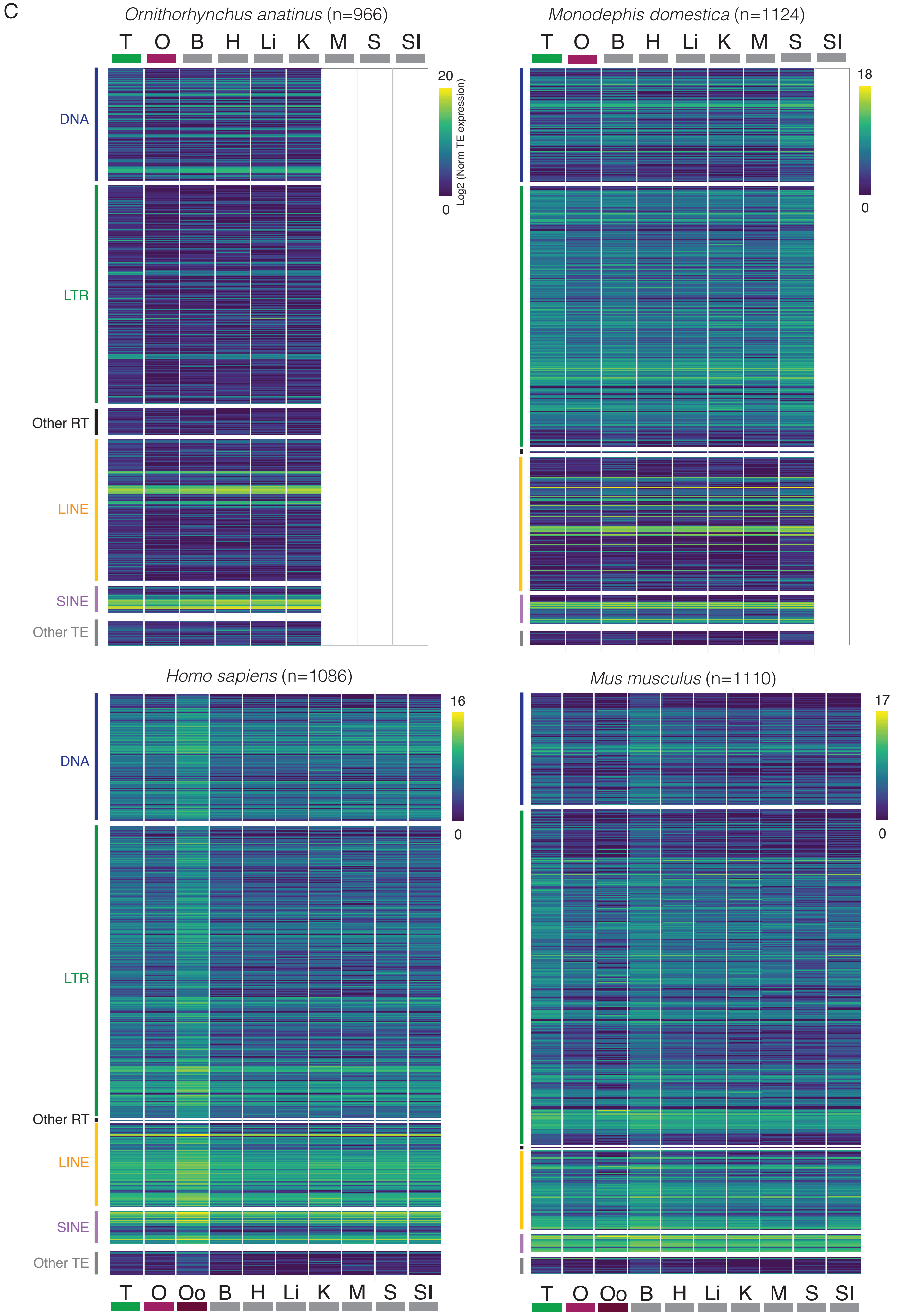

Supplementary fig. S6C. Total-TE derived transcript expression across tissues. Heatmaps show that individual TE expression levels across tissues are more variable in mammal species compared to other vertebrates, and highlight how pervasive transcription of TEs represents a common feature of healthy tissue transcriptomes. It is also noticeable that only in humans the oocytes have higher total-TE derived transcript levels than testes.

A

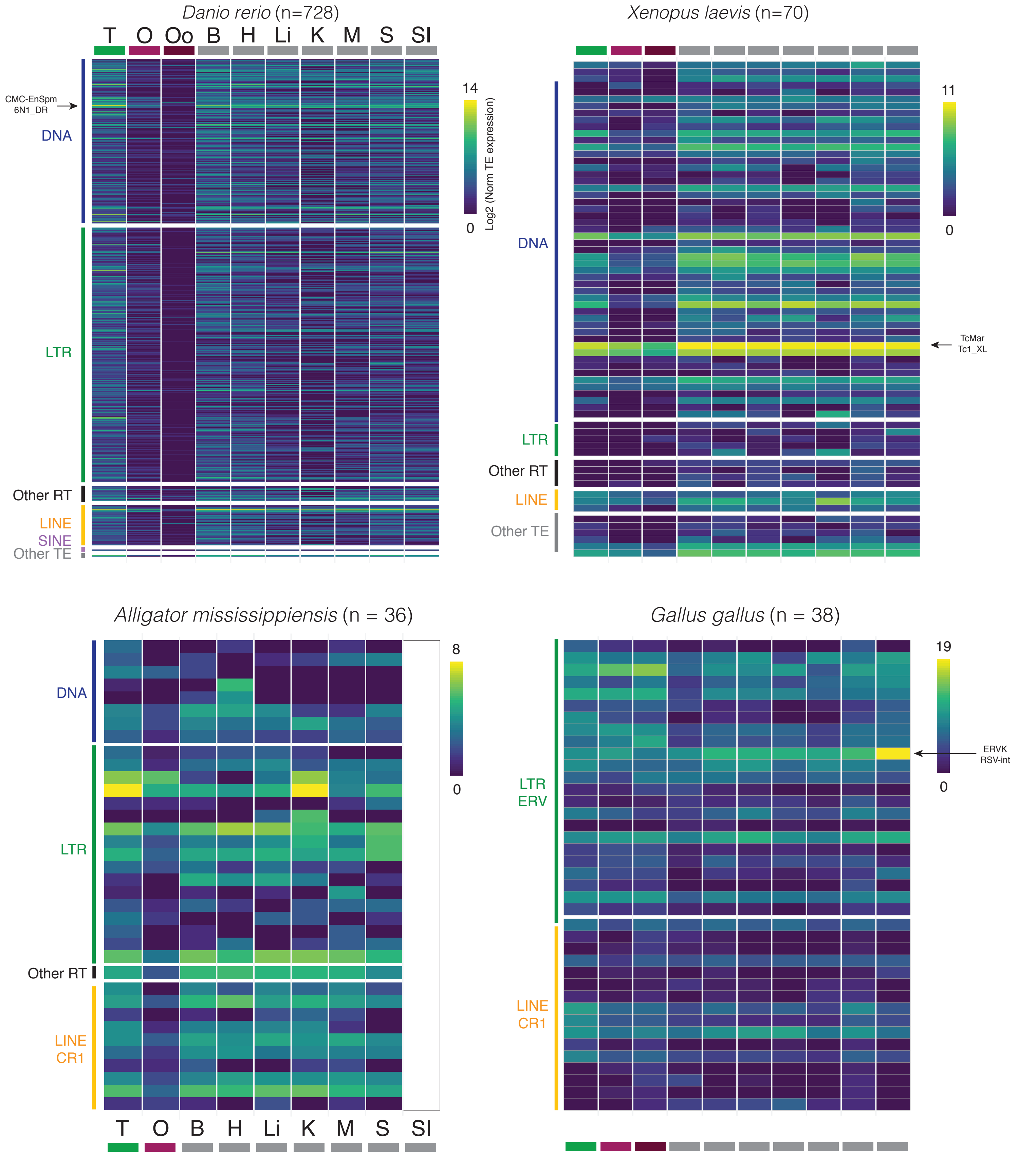

Supplementary fig. S7A. Recent-TE derived transcript expression across tissues. Heatmaps show individual TE expres-sion levels (rows) across tissues (columns) for lower vertebrates and archosauria reptiles. Empty columns represent tissues with unavailable transcriptome data. Hearmaps also reflect the relative abundance in terms of number of individu-al elements belonging to each of the major TE classes. T = testis; O = ovary; Oo = oocyte; B = brain; H = heart; Li = liver; K = kidney; M = muscle; S = spleen; SI = small intestine. LTR = Long Terminal Repeats; Other RT = other retrotrans-posons (PLE and DIRS); LINE = Long Interspersed Nuclear Elements; SINE = Short Interspersed Nuclear Elements. Across vertebrates, the zebrafish (*Danio rerio*) is one of the few species to have remarkably high incidence of recent-TEs (n= 728; generated from genomic loci with a Kimura 2D distance from the consensus less than 2%) that include all major TE families, supporting high genomic turnover of TEs in the species. On the other hand, most vertebrate species are characterized by a very small number of recent-TEs that generate transcripts. Arrows on the margin provide examples of highly expressed TEs.

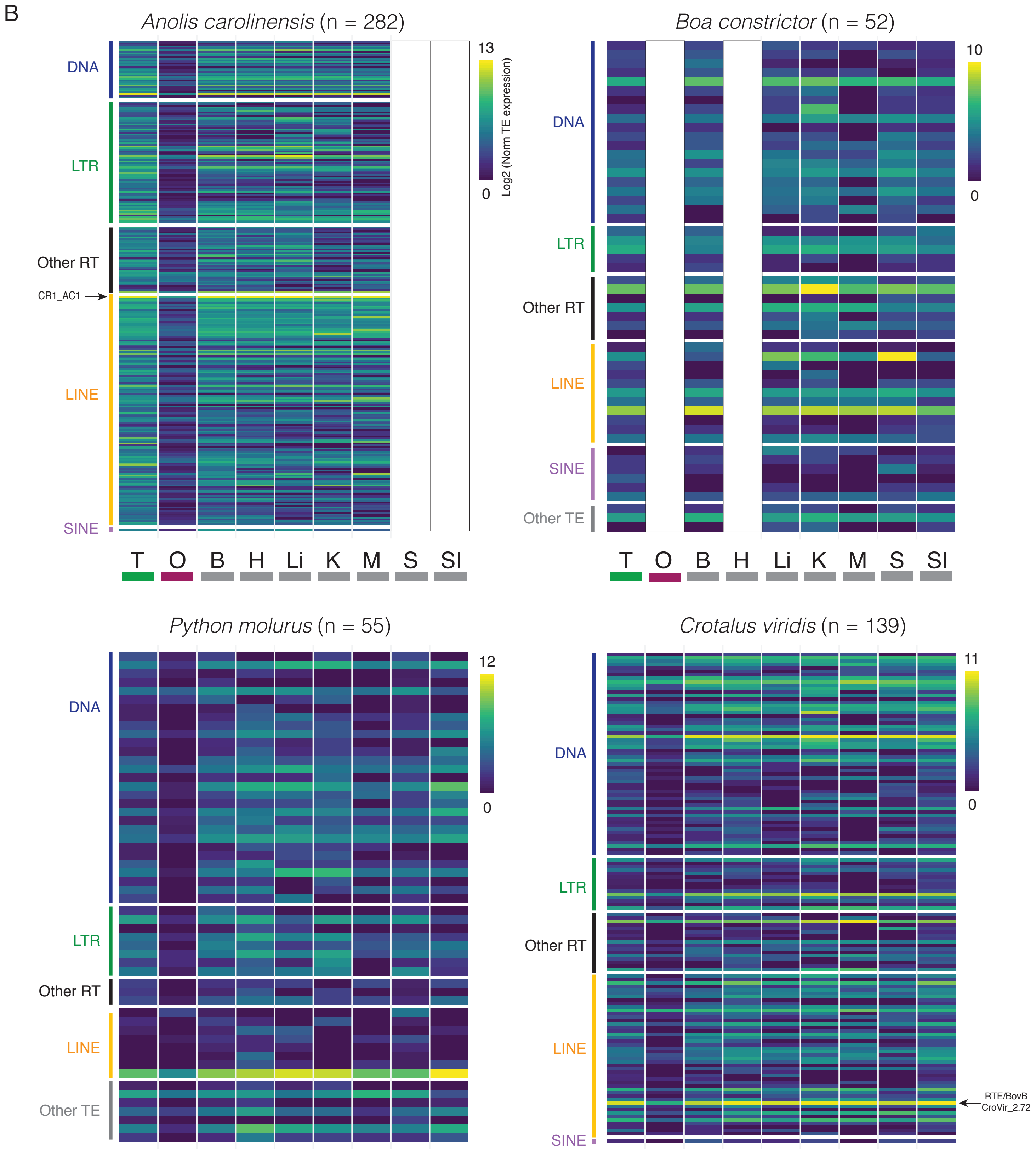

Supplementary fig. S7B. Recent-TE derived transcript expression across tissues in squamate reptiles. Heatmaps show individual TE expression levels (rows) across tissues (columns) of TEs that recently amplified in squamate genomes. Among squamates, the two non-colubroid snake species (*Boa constrictor* and *Python molurus*) show only a small number of TEs (although belonging to several subfamilies) being capable of originating transcript, in contrast to the green anole lizard and the prairie rattlesnake that show more highly dynamic TE transcriptomes. Arrows on the margin provide examples of highly expressed TEs.

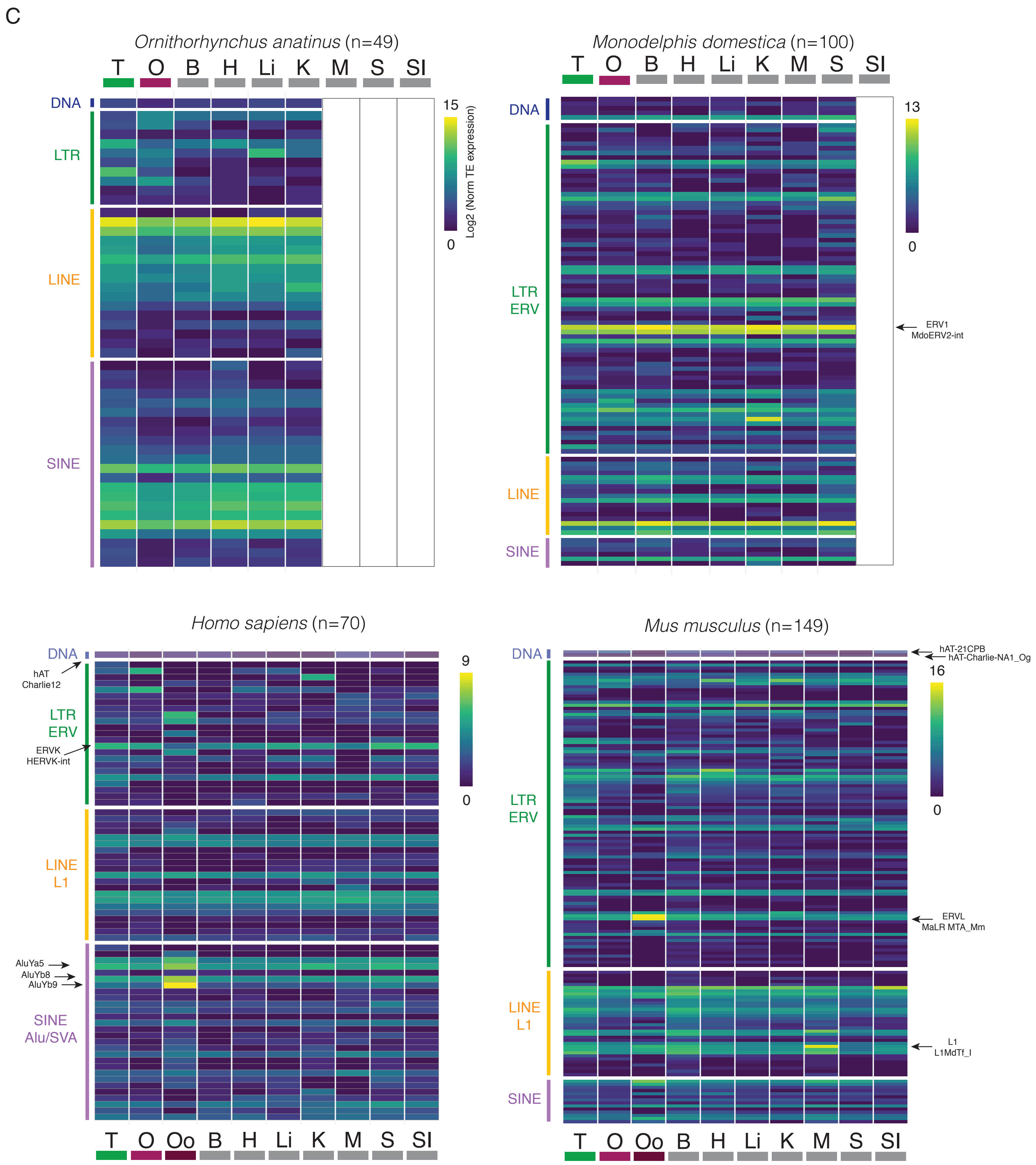

Supplementary fig. S7C. Recent-TE derived transcript expression across tissues in mammals. Heatmaps show individ-ual TE expression levels (rows) across tissues (columns) of recently expanded TEs across the mammalian radiation. Compared to other vertebrate species, mammals are characterized by a compelling small number of families (mostly L1 and SINEs in theria, L2 LINEs in the platypus, and ERV LTRs in the opossum) capable to originate transcripts. Surprisingly, we found also few DNA elements (faded in human and mouse heatmaps) in our subsample of recent-TE inserts, which might either reflect incorrect mapping/attribution, or the result of transcriptional read-through or of pervasive transcription. Arrows on the margin provide examples of highly expressed TEs.

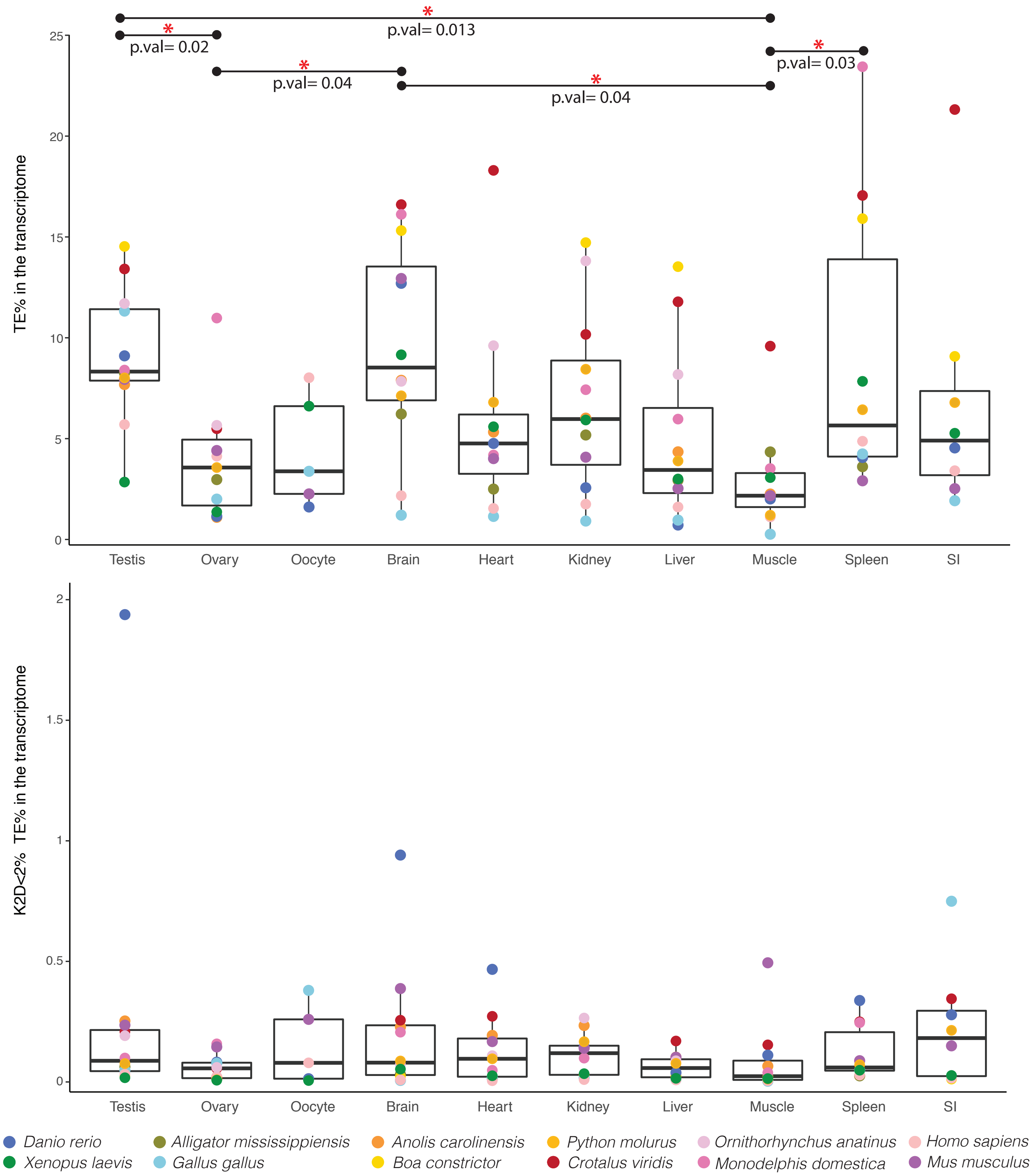

Supplementary fig. S8. Contribution of TEs to the transcriptome of germline and somatic tissues. Box plots illustrate the variation across species in the percent of the transcriptome (following within species normalization across tissues) corresponding to annotated TE-derived transcripts (top) and to transcripts derived from recently amplified TEs (bottom). Analysis of TE transcripts between tissues for 12 vertebrate species highlights remarkable variance in TE expression levels both across species and among tissues per each species. However, we find variability to be much lower when only recent-TEs are analyzed (indeed, there is no significant difference in average expression between tissues), suggesting pervasive transcription of transpositionally incompetent TEs in vertebrate tissues. Thick black bars in the box plots represent median TE% in the transcriptome for each tissues, and top and bottom margins the first and third quartile of the distribution. We performed multiple pairwise comparisons between groups (pairwise Wilcoxon test following a Kruskal-Wallis rank sum test across tissues) to test if average TE expression values were significantly different between tissues (only significant results are shown).

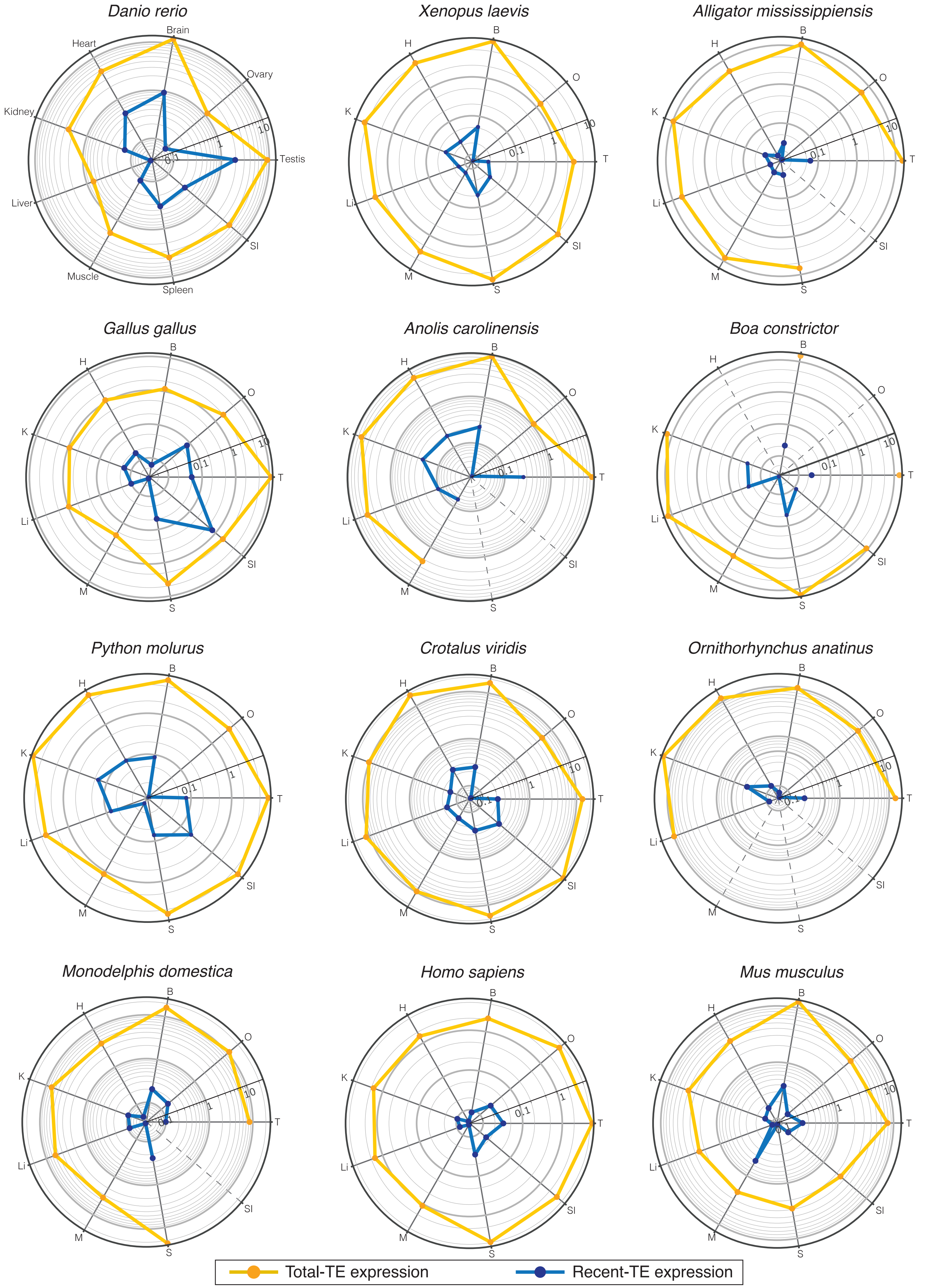

Supplementary fig. S9. Transcriptome estimates of total and recent TE expression levels. Radar plots show the percentage of the transcriptome (on a log10 scale) made up by total-TE transcripts (total-TEs; yellow) and by recently inserted TE copies (recent-TEs; blue) across somatic and germline tissues. We found abundant TE tran-scription to be a common physiological feature across all vertebrate tissues, whereas recent-TEs represent a marginal fraction of the transcriptomes.

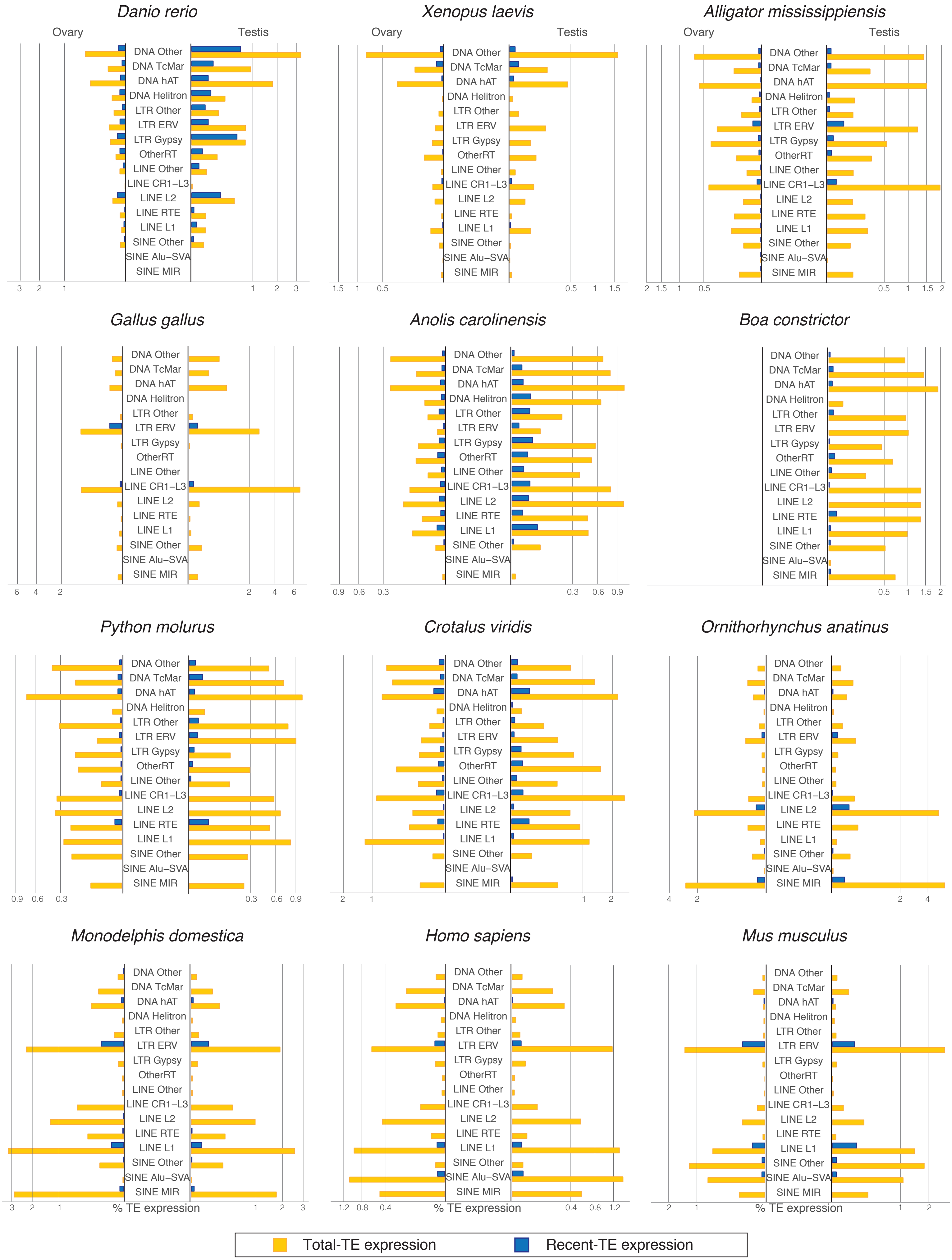

Supplementary fig. S10. Expression level estimates of total and recent major TE families expression levels in germline tissues. For each species, barplots show the difference between total (yellow) and recently inserted TE copies (blue) expression level estimates in ovary (left) and testis (right) transcriptomes. Expression levels are reported as transcriptome percent and plotted in logarithmic scale.

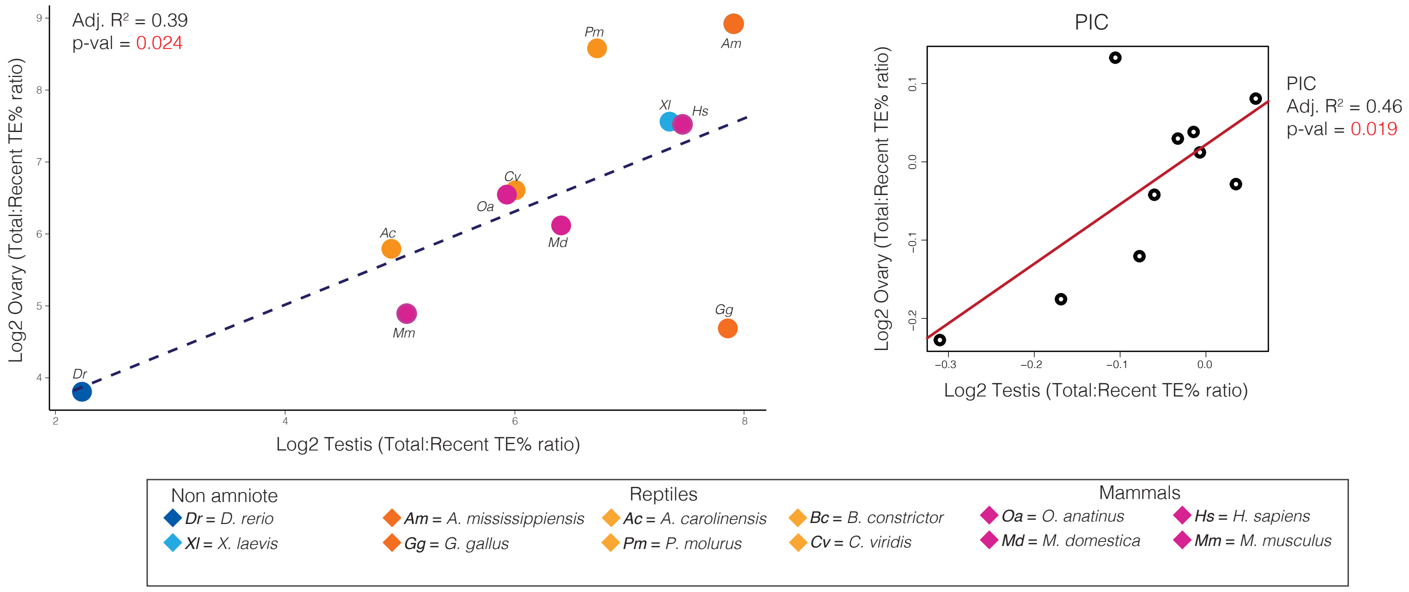

Supplementary fig. S11. Relationships between total and recent TE expression levels in germline tissues. Scatterplot and phylogenetically independent contrast (PIC) show a positive exponential correlation between the fold change in percentages of total-TE transcripts and recent-TE transcripts between testis and ovary across vertebrate species.

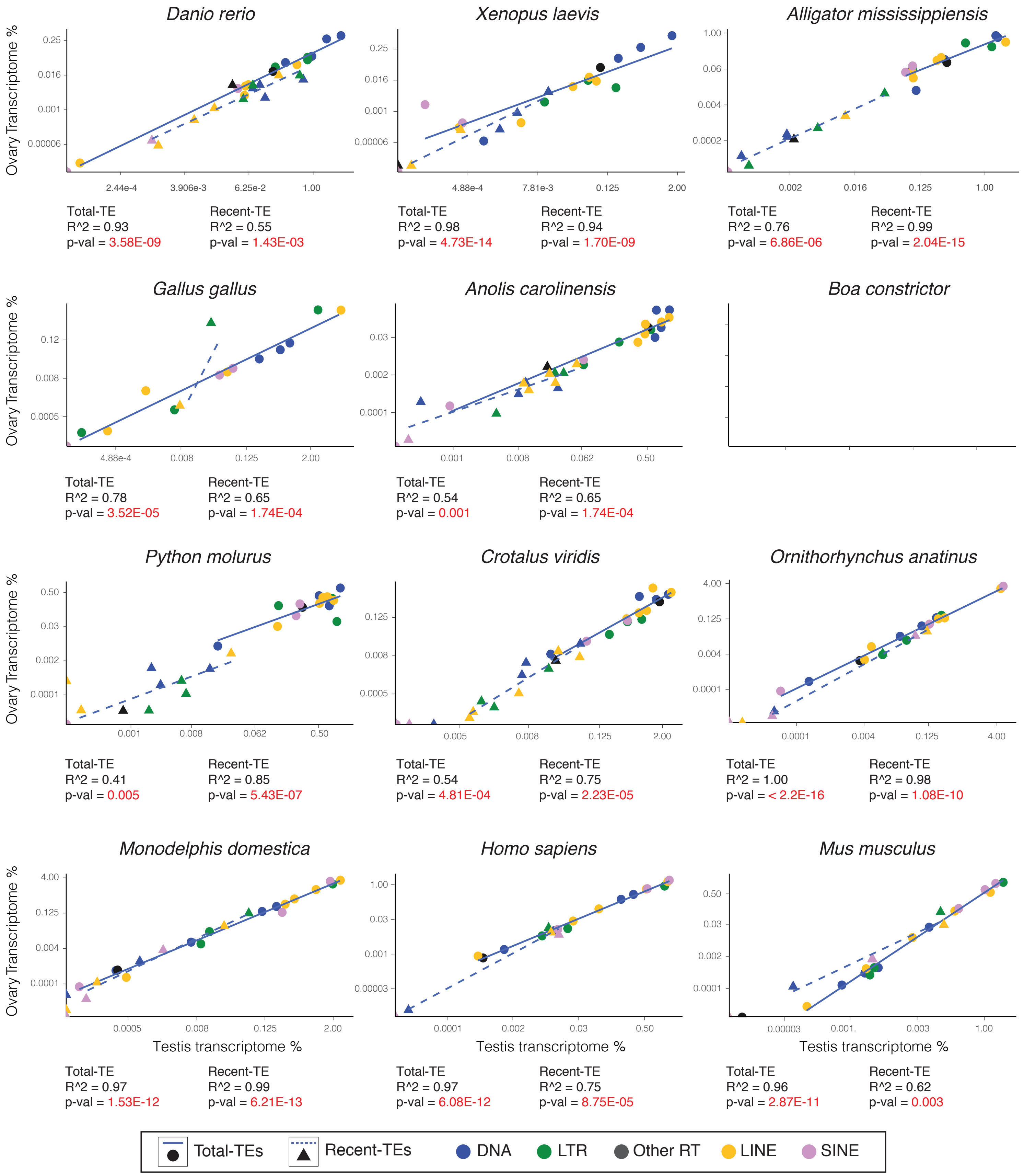

Supplementary fig. S12. Regression analyses of TE expression levels in germline tissues across vertebrate species. Scatterplots show positive correlative trends of major TE subfamilies relative composition between the male and female germline in the total-TE (circles and solid lines) and recent-TE (triangles and dashed lines) datasets. Regression analyses were performed on transcriptome percent values. Scatterplots axes have been log2 transformed for display purposes. Reptile species display a stronger association (higher R^2 values) between germline tissue expression levels of TE subfamilies when only recent-TEs are considered compared to other vertebrate species, that show the opposite trend instead.

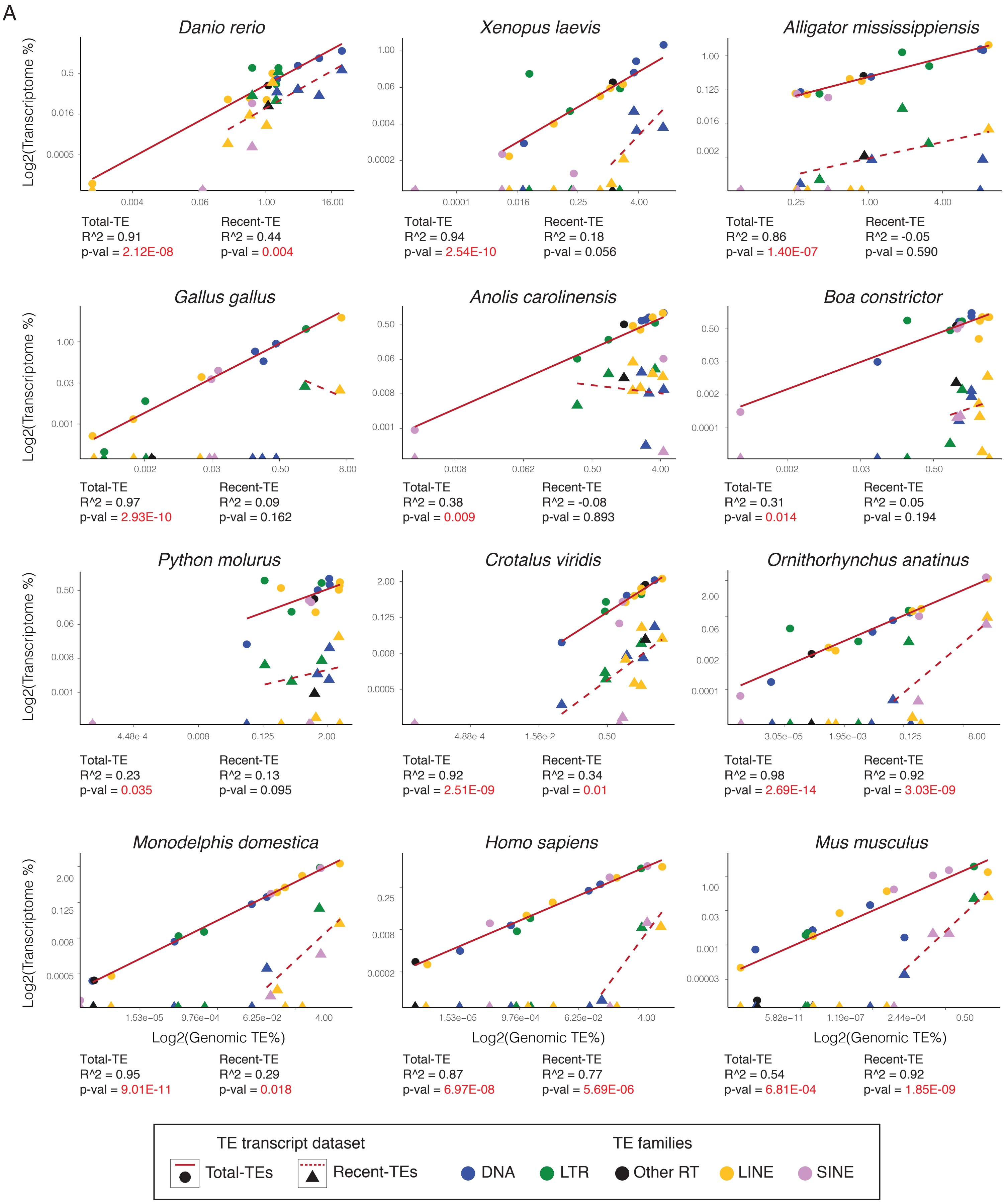

Supplementary fig. S13A. Regression analyses of TE expression levels and genomic TE content in germline tissues across vertebrate species. A) Scatterplots show positive correlative trends of major TE families relative composition in the male germline between total transcriptome and genomic content (total-TEs; circles and solid lines), supporting a pervasive model of TE transcription. In contrast, linear regressions between relative composi-tion of the total TE genomic content and recent-TE transcripts (triangles and dashed lines) show absence of a relationship in most non-mammal species, and a positive, although weaker correlation across mammals (and zebrafish and prairie rattlesnake). Regression analyses were performed on transcriptome percent and genomic percent values. Scatterplot axes have been log2 transformed for display purposes.

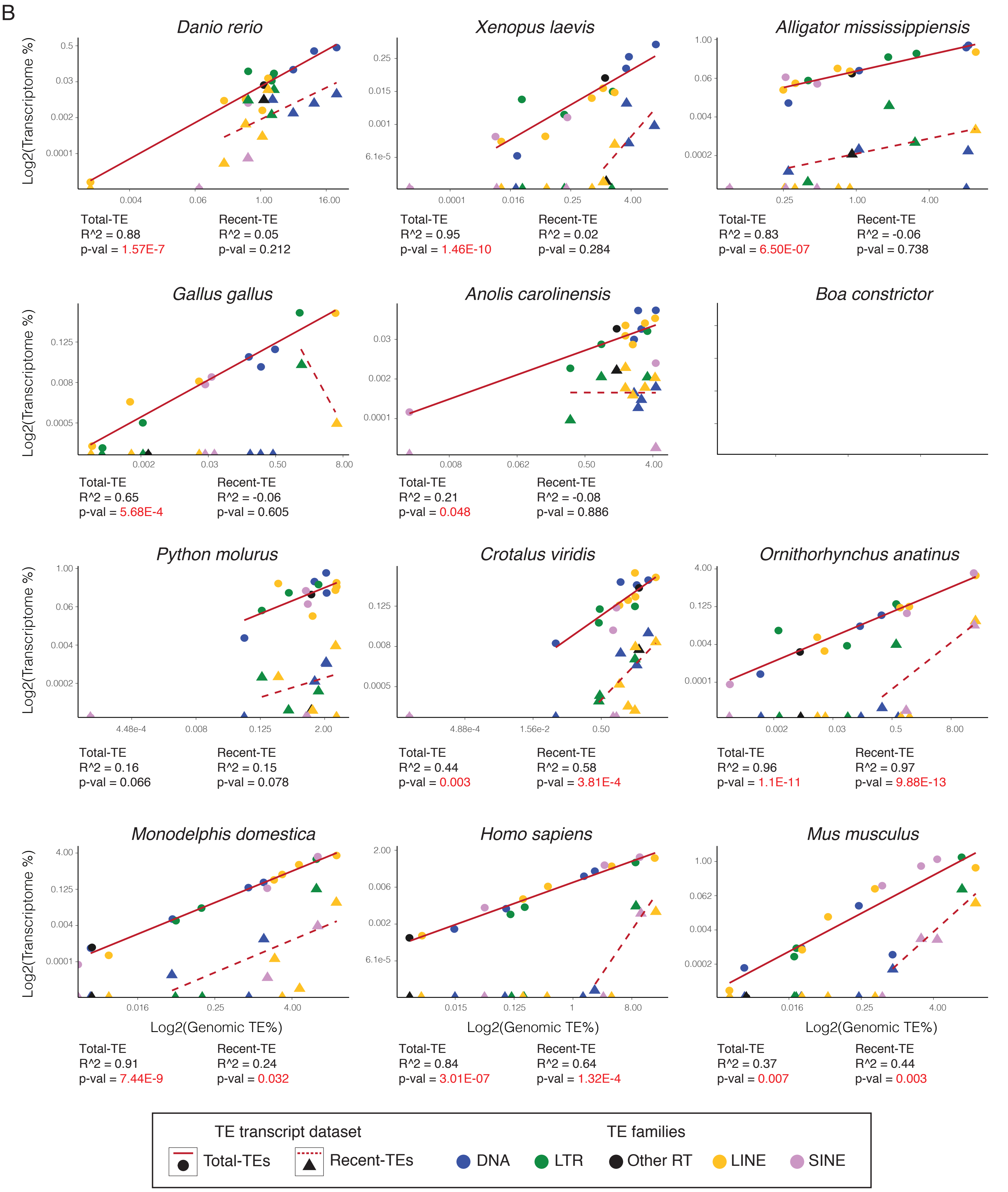

Supplementary fig. S13B. Regression analyses of expression levels and genomic TE content in germline tissues across vertebrate species. B) Scatterplots show positive correlative trends of major TE families relative composition in the female germline between total transcriptome and genomic content (total-TEs; circles and solid lines), supporting a pervasive model of TE transcription. In contrast, linear regressions between relative composition of the total TE genomic content and recent-TE transcripts (triangles and dashed lines) show absence of a relationship in most non-mammal species, and a positive, generally weaker, correlation across mammals (and prairie rattlesnake). Regression analyses were performed on transcriptome percent and genomic percent values. Scatterplot axes have been log2 transformed for display purposes.

Supplementary fig. S14. Expression levels of recent-TEs, TE regulatory pathways, and their correlations. A) Heatmaps show expression levels of recent-TEs and TE negative regulatory pathways in germline and somatic tissues across vertebrate species. While germline tissues show the highest proportional activation of TE silencing mechanisms, the spleen stands out among somatic tissues for high expression of recent-TEs, transcriptional and post-transcriptional regulatory mechanisms. B-D) Phylogenetic independent contrast (PIC) Spearman rank-order correlation analyses across vertebrate species suggest a non significant positive correlative trend between TE expression and transcriptional regulators (Tr) across tissues, and a negative trend with PIWI pathway genes in somatic tissues. Although not significant, we detect a positive correlative trend between TEs and regulators expression in the testis, whereas the opposite is found in the ovary.

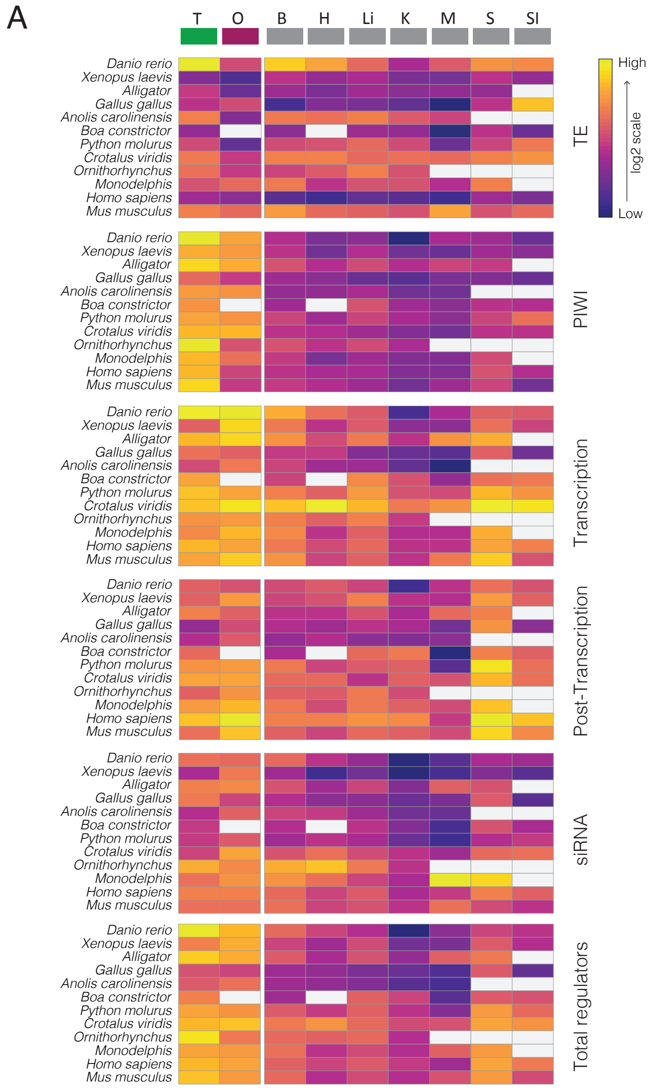

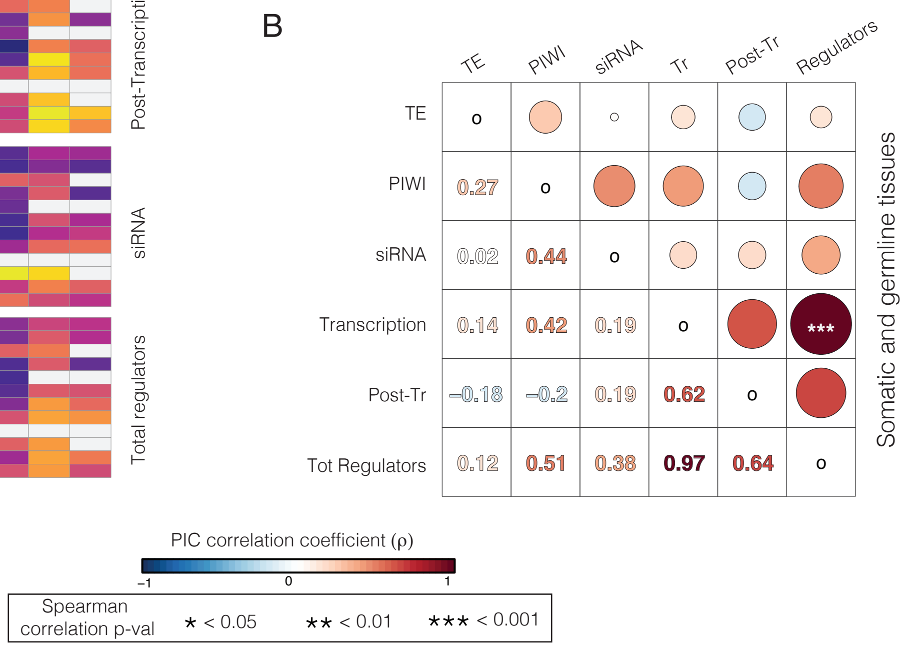

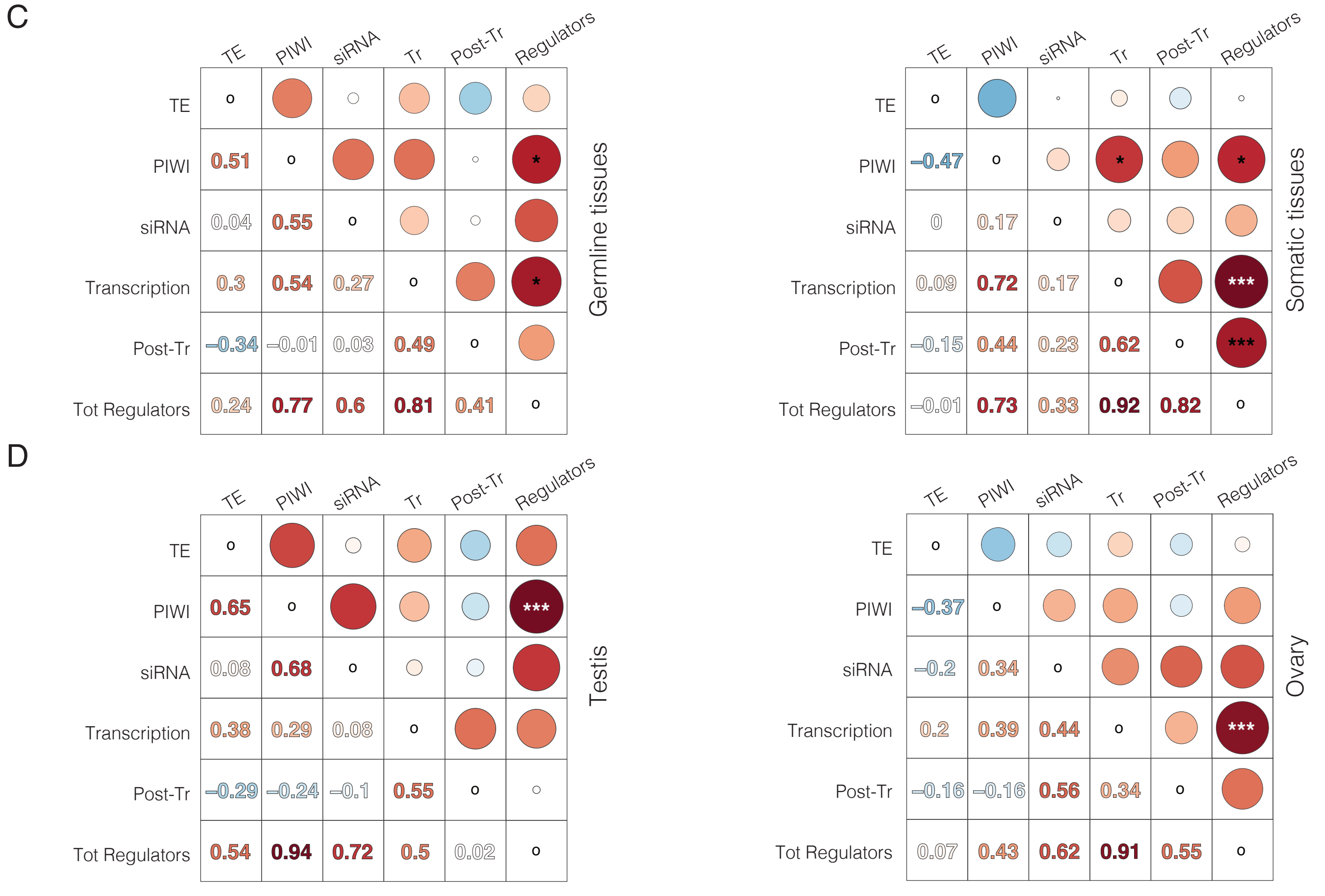

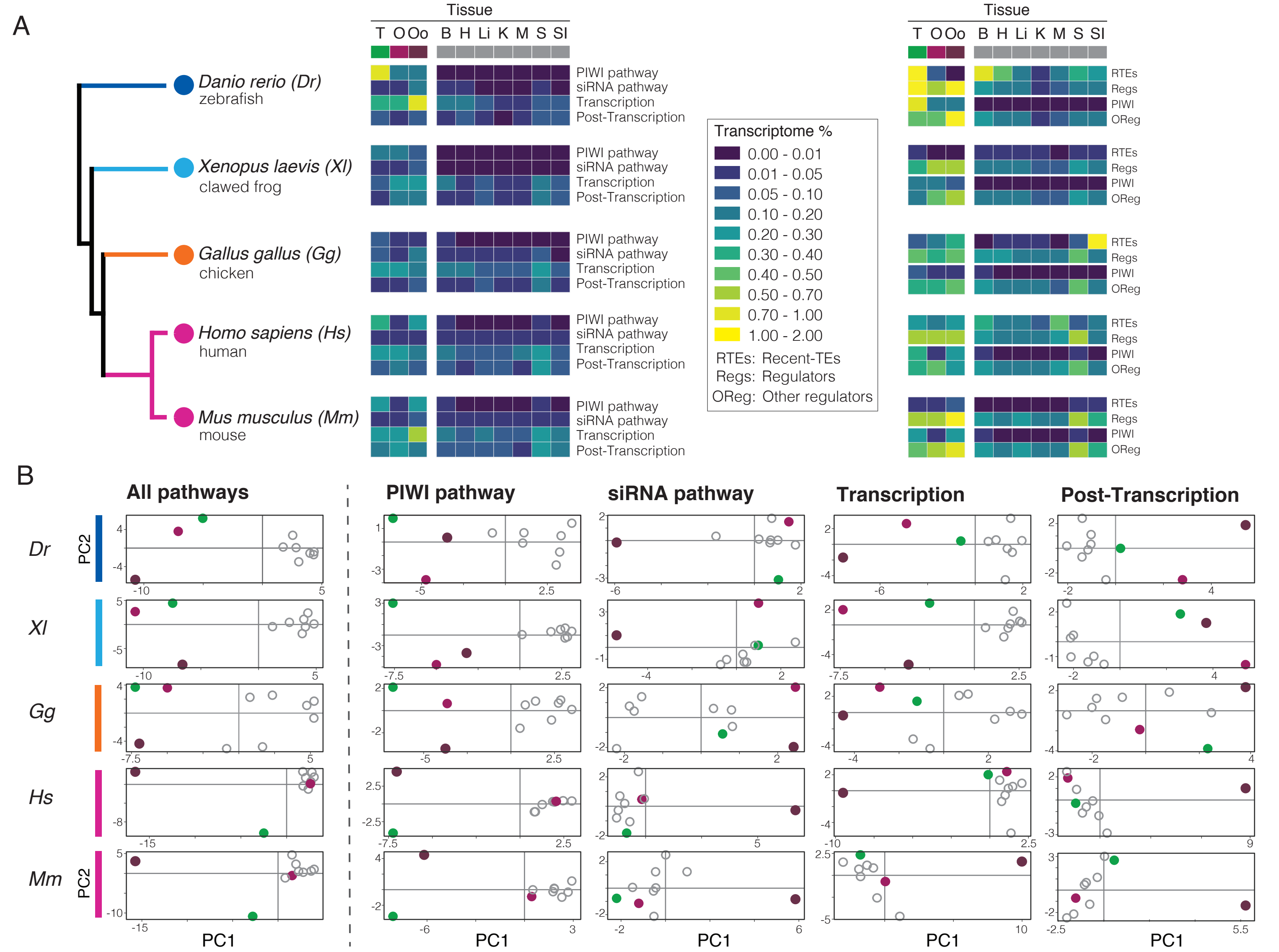

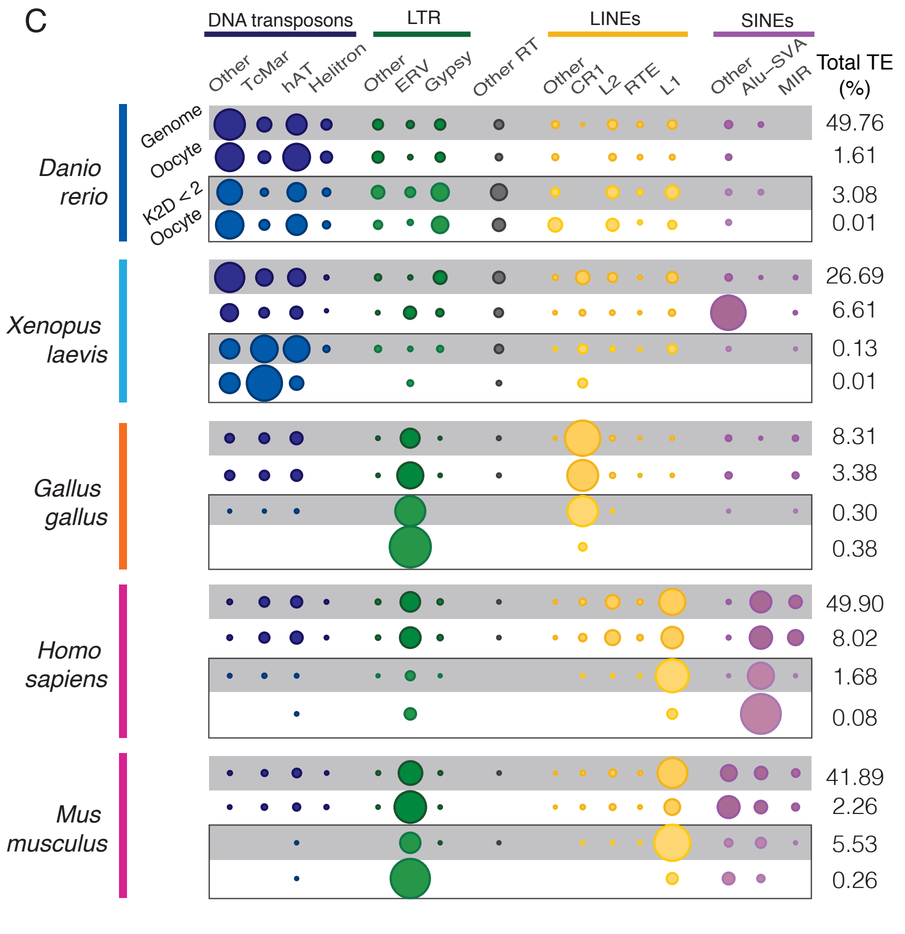

Supplementary fig. S15. Negative TE regulators and TE-derived expression profile in oocyte samples. (A) For five vertebrate species, heat maps show expression profiles of key genes involved in TE negative regulatory mechanisms across germline tissues (T= testis; O= ovary), oocytes (Oo), and somatic tissues, and how they relate to recent TE-derived transcript expression. Comparative expression levels are reported as percentage of the transcriptome following within species normalization. (B) Principal component analyses (PCAs) for total and individual main TE regulatory mechanisms reflect variance in gene expression profiles across tissues for each species. Oocytes (burgundy) are consistently discriminated from other germline and somatic tissues in all species analyzed, with variance in expression patterns being remarkable in mammalian species. (C) Relationship between genomic and transcriptomic TE relative composition in oocyte cells. Area of the circles in the balloon plot reflects the percentage of major TE subfamilies-relative to the total genomic TE content (top row, grey background) and to the total TE transcriptome (white background). In the box, the same relationship is displayed for recently inserted TE copies (with a Kimura distance < 2%) and recent-TEs in the transcriptome. Values to the left report the real proportion of TEs (TE content %) in the genomes and transcriptomes.

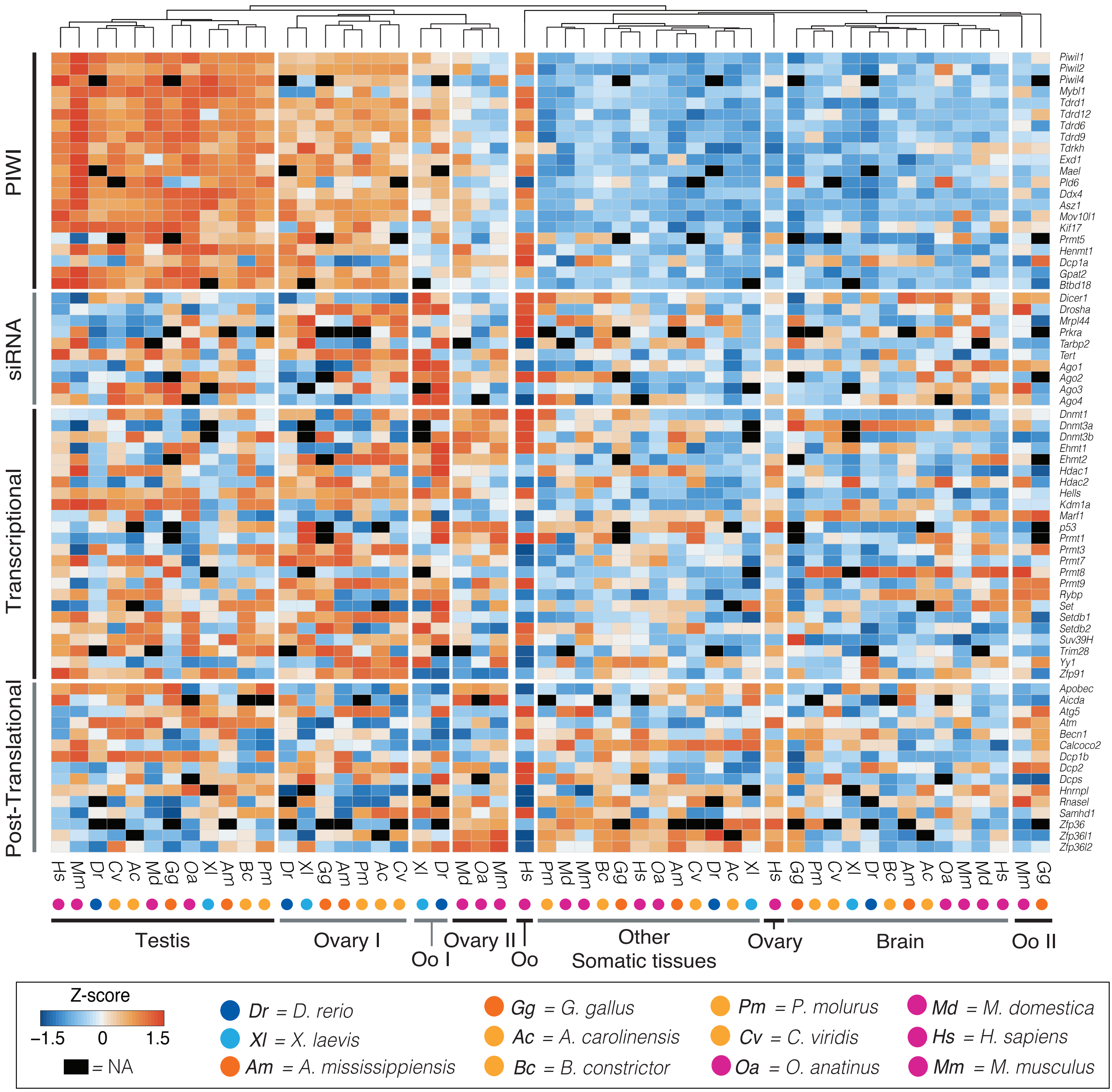

Supplementary fig. S16. Hierarchical clustering Z-score heatmap of TE regulatory genes in germline and somatic vertebrate tissues. Analysis of differential expression of key conserved genes involved in TE silencing suggests the existence of 5 main expression profiles across vertebrate tissues: vertebrate testis, characterized by the highest activation status of the PIWI:piRNA pathway and transcriptional regulators; ovary of non-mammal species, with expression patterns similar to the testis; mammalian ovary (to the exclusion of humans), which shows a sharp decreased expression of PIWI genes; other somatic tissues (average Z-scores across heart, kidney, liver, muscle, spleen and small intestine after individual tissue heatmap supported the existence of a single cluster); and vertebrate brain. Oocyte cells (Oo) of zebrafish (*D. rerio*), clawed frog (*X. laevis*) and human (*H. sapiens*) show expression profiles of PIWI-pathway genes similar to those of the ovary of non-mammal species, and comparatively higher activity of the siRNA pathway. Oocytes of mouse (*M. musculus*) and chicken (*G. gallus*) show instead more similarities in TE regulatory mechanisms expression profiles with the brain and other somatic tissues.

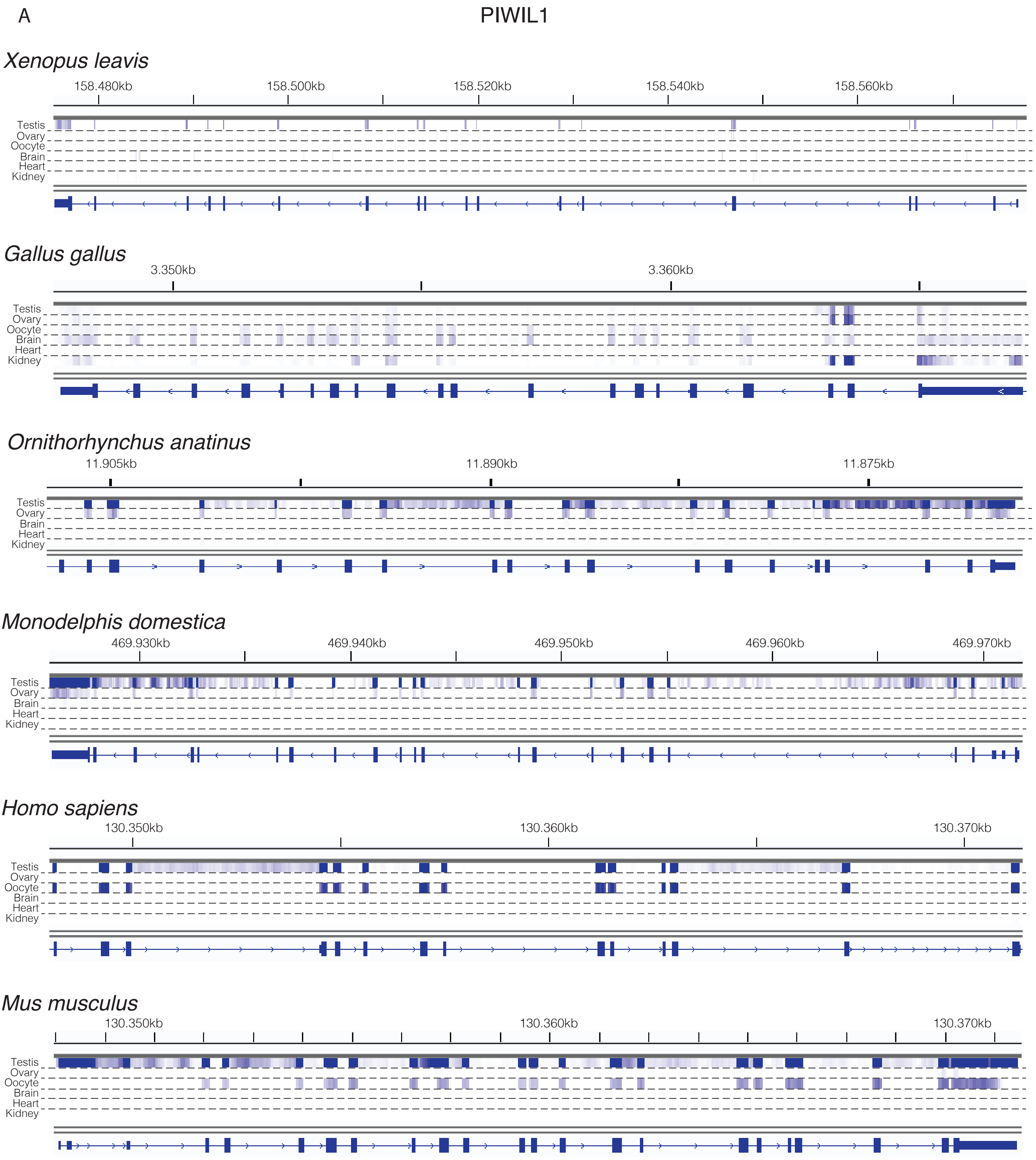

Supplementary fig. S17. Read coverage tracks of three conserved *PIWI* genes for a subsample of 6 vertebrate species. Trakcs were generated by converting STAR sorted bam output files to bigwig files (at a default resolution of 50bp). Bigwig files were then imported into IGV and visualized in the context of the genome and gtf files used to align reads in STAR; multimapping reads were retained in the final alignment as required by TEtranscript. Read coverage is visualized as haetmaps of log2 scaled coverage values, with darker blue representing higher coverage and lighter blue lower coverage. Read coverage was not autoscaled across tissues for a species. (A) Heatmap of PIWIL1 read coverage. Expression of PIWIL1 is consistently highest in testes across vertebrates, followed by the female germline (ovary in platypus and opossum; oocytes in chicken, human and mouse). For the clawed frog, a second PIWIL1 orthologue has been described (PIWIL1.s - not shown), characterized by female germline expression bias, whereas PIWIL1.L has male germline expression bias. For most species expres-sion of PIWIL1 is limited to the germline, yet in some species (e.g., chicken), it appears to be also expressed in somatic tissues.

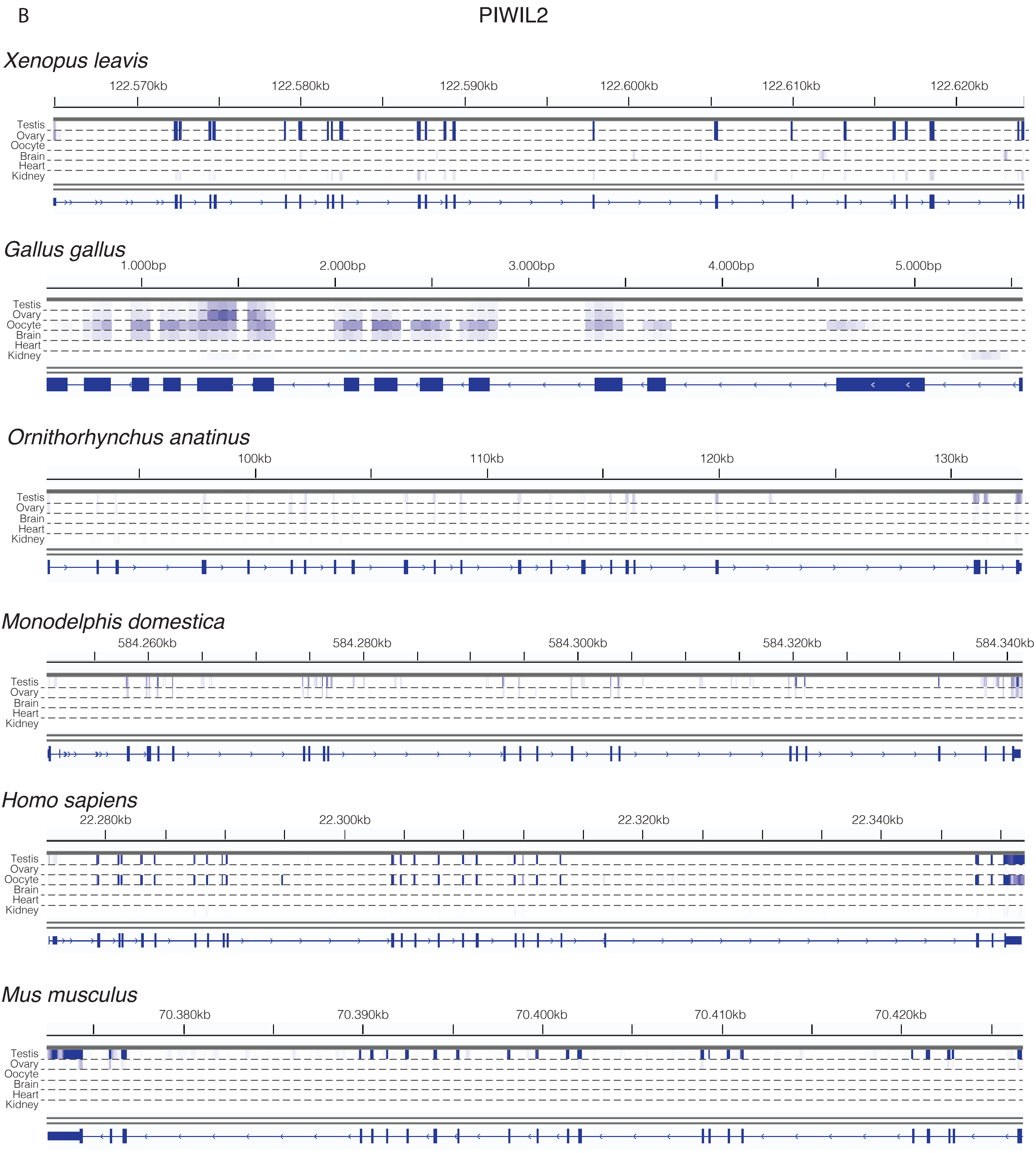

Supplementary fig. S17B. Read coverage tracks of the *PIWIL2* gene for a subsample of 6 vertebrate species. Trakcs were generated by converting STAR sorted bam output files to bigwig files (at a default resolution of 50bp). Read coverage is visualized as haetmaps of log2 scaled coverage values, with darker blue representing higher coverage and lighter blue lower coverage.

Expression of PIWIL2 is consistently highest in testes across vertebrates, followed by the female germline (ovary in platypus and opossum, but not in clawed frog oocytes; oocytes in chicken and human, but not in mouse). For most species expression of PIWIL2 is limited to the germline, yet in some species (e.g., clawed frog, chicken and platypus), it appears to be also expressed at lower levels in somatic tissues.

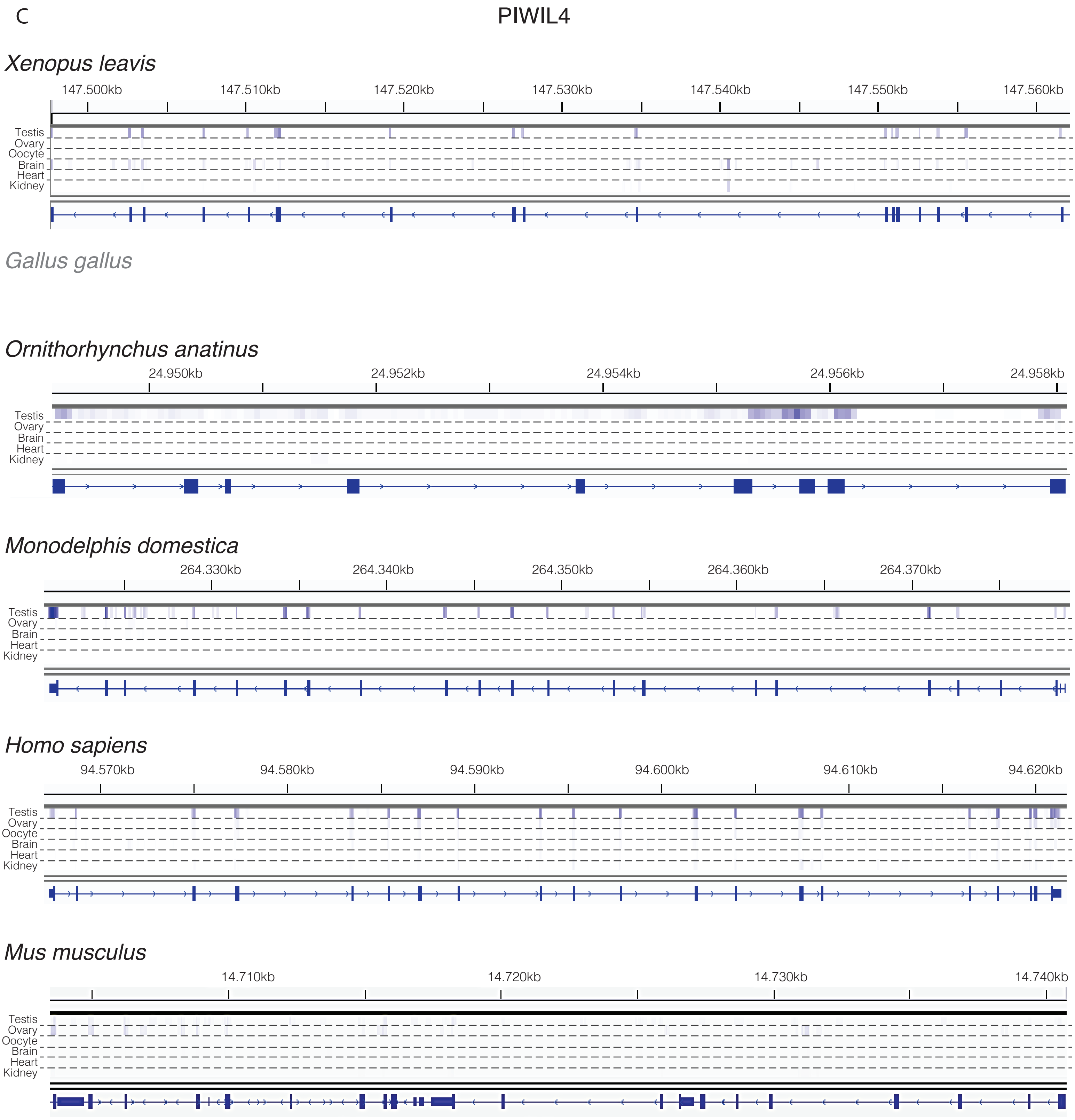

Supplementary fig. S17C. Read coverage tracks of the *PIWIL4* gene for a subsample of 5 vertebrate species. Trakcs were generated by converting STAR sorted bam output files to bigwig files (at a default resolution of 50bp). Read coverage is visualized as haetmaps of log2 scaled coverage values, with darker blue representing higher coverage and lighter blue lower coverage.
Expression of PIWIL4 across tissues is comparatively lower than expression of PIWIL1 and PIWIL2 whithin species; with a single exception (the clawed frog), expression also seems to be limited to the male germline.

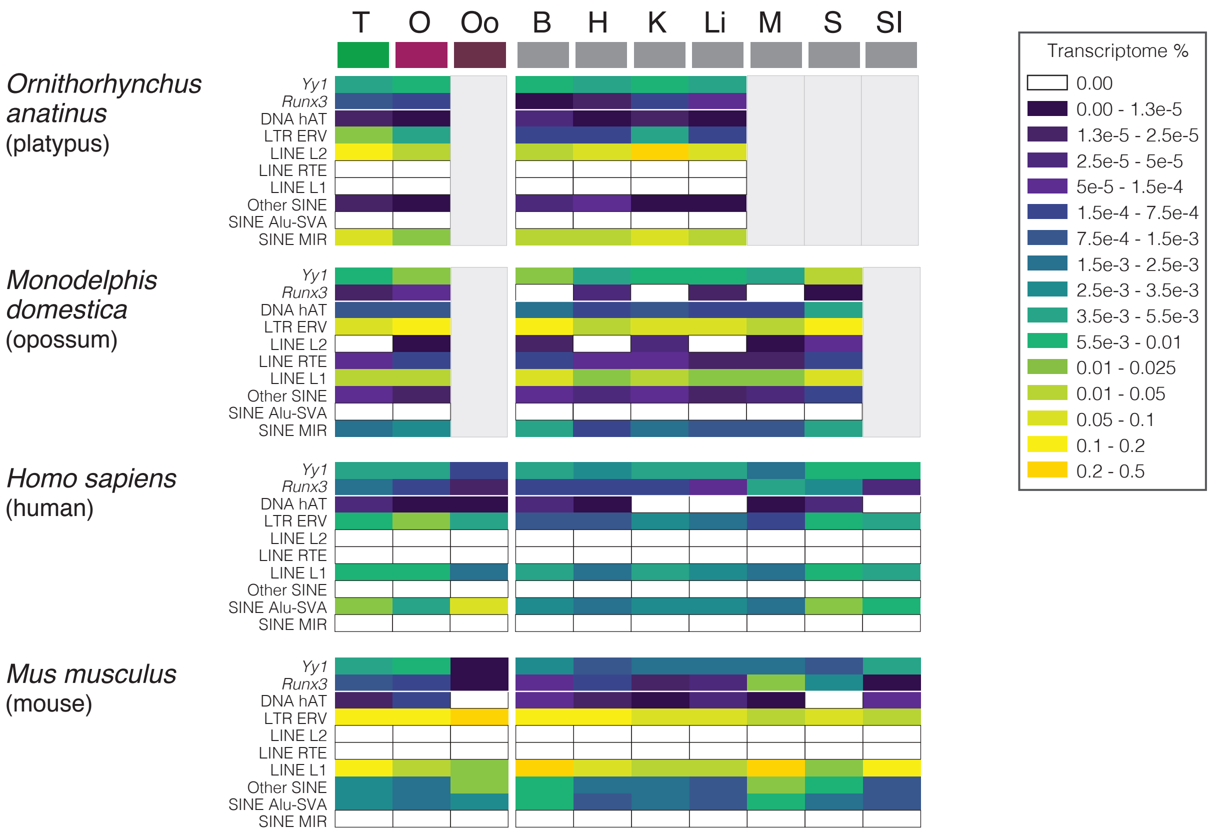

Supplementary fig. S18. Relationships between recent TE-derived transcripts and positive regulators of TE expression in mammalian germline and somatic tissues. *Yy1* is an ubiquitous, multi-functional transcription factor that in a context-specific way can either promote or repress gene and TE expression. Runx3 is another transcription that can bind TE internal promoters to direct their expression. Although both tran-scription factors have a broad target range, we sought to investigate if *Yy1* and *Runx3* expression levels could show similar profiles of recent-TE expression levels across multiple somatic and germline tissues. Across human tissue, where activity of *Yy1* and *Runx3* has been more extensively characterized, our analyses are broadly in line with the current literature, as profiles of expression levels of the two transcrip-tion factors resemble more expression profiles of L1 LINEs than those of other TE families. In mouse, binding sites for *Runx3* in L1 promoters have not yet been identified, contrarily to *Yy1* binding sites. Our analyses partially agree with the literature, as expression levels of L1s (or of any another TE family) across sampled tissues appear unrelated to those of either *Runx3* or *Yy1* . However, given the context-specific activity of both transcription factors and the complexity of TE regulation, we cannot reject that *Runx3* and/or *Yy1* are indeed capable of regulating TE activity. We included analyses of other two representatives of the mammalian radiation, platypus and opossum for comparative purposes. We were not able to identify any relationship between TE-derived transcripts and expression levels of *Yy1* and *Runx3*, yet we can not completely discard the possibility that the two transcription factors may regulate different subsets of TEs in different contexts than in human and mouse.
