# Supplementary material for: Vertebrate Lineages Exhibit Diverse Patterns of Transposable Element Regulation and Expression across Tissues
Source: Genome Biol Evol. 2020 Apr 9;12(5):506–21. doi: 10.1093/gbe/evaa068 (PMC7211425; doi:10.1093/gbe/evaa068)
Supplement: evaa068_Supplementary_Data [file evaa068_supplementary_data.zip › Supplemetary_Methods.docx]

Transposable element regulation and expression varies across tissues between major vertebrate lineages

Giulia I.M. Pasquesi^1,2^, Blair W. Perry^1^, Mike W. Vandewege^3^, Robert P. Ruggiero^4^, Drew R. Schield^1,5^, and Todd A. Castoe^1 ‡^

## Supplemental methods

In this study we used previously published poly-A-selected RNAseq datasets that are available on the NCBI SRA archive database ([Leinonen, et al. 2011](#_ENREF_15); Supplementary file S1). Raw RNAseq data were first filtered for prokaryote and eukaryote rRNA transcripts using *SortMeRNA* v2.1 ([Kopylova, et al. 2012](#_ENREF_14)), and then quality and adapter trimmed in *Trimmomatic* 0.36 ([Bolger, et al. 2014](#_ENREF_3)). We used *FastQC* v0.11.8 ([Andrews 2010](#_ENREF_2)) to assess quality of the processed reads. Since quality assessment passed analyses for all parameters without warnings, no further read filtering was performed. Forward and reverse paired reads from *Trimmomatic* filtering were then used as input for estimation of gene and transposable element (TE) expression levels.
For each species, we used *STAR* v2.7.0f ([Dobin, et al. 2013](#_ENREF_7)) to map reads to the latest genome version and annotation .*gff* files available on the NCBI Genome database ([Sayers, et al. 2019](#_ENREF_18)) at the time of analyses (*Danio rerio*: assembly GRCz11, *Xenopus laevis*: assembly Xenopus_laevis_v2, *Alligator mississippiensis*: assembly ASM28112v4, *Gallus gallus*: assembly GRCg5, *Anolis carolinensis*: assembly AnoCar2.0, Boa constrictor: ERS218597, snake_7C available on GigaDB ([Bradnam, et al. 2013](#_ENREF_4)), *Python molurus*: assembly Python_molurus_bivittatus-5.0.2, *Crotalus viridis*: assembly UTA_CroVir_3.0, *Ornithorhynchus anatinus*: mOrnAna1.p.v1, *Monodelphis domestica*: assembly MonDom5, *Homo sapiens*: assembly GRCh38.p12, *Mus musculus*: assembly GRCm38.p6). For human and mouse, primary genome assemblies and corresponding annotation files were used to avoid incorrect read alignment to loci on patches or alternative haplotype contigs as suggested by the authors ([Dobin, et al. 2013](#_ENREF_7)). *STAR* was run according to default parameters but using the variables *-winAnchorMultimapNmax 100* and *-outFilterMultimapNmax 100*, which allow for multiple alignments of maximum 100 reads, as specified in [Jin, et al. (2015](#_ENREF_12)).

Expression levels were estimated using *TEtranscript* ([Jin, et al. 2015](#_ENREF_12)), a tool that allows for the simultaneous estimation of gene and TE expression levels. To assign mapped reads to a genomic locus, *TEtranscript* leverages two annotation files that specify gene and repeat element coordinates, respectively. We used the same gene annotation files provided as a reference when running *STAR* to build the gene index structure; to convert *.gff* files into the required *.gtf* files we used the *gff3ToGenePred* and *genePredToGtf* modules available on the UCSC website ([Casper, et al. 2018](#_ENREF_6)). TE index structures were built from *RepeatMasker* ([Smit 2013-2015](#_ENREF_20)) runs; for all species, we used the standard tetrapoda library as reference, except for human and mouse for which we used the mammal library. For snake species, we used snake-specific libraries in combination with the standard tetrapoda library following the same strategy described in ([Schield, et al. 2019](#_ENREF_19)) instead. We used the script *makeTEgtf.pl* available made available from the Hammell lab (http://labshare.cshl.edu/shares/mhammelllab/www-data/TEToolkit/TE_GTF/makeTEgtf.pl.gz) to convert RepeatMasker .*out* files into *.gtf* files. *TEtranscript* was run using default parameters, using the *--multi* flag and specifying whether transcriptome data was stranded or not. To further focus our analyses on TE-derived reads that originated from recently active TE copies (and thus more likely targeted by repressive mechanisms; Sun, et al. 2017.), in a second, separate analysis we provided *TEtranscript* with a .*gtf* annotation file containing only TE loci that according to the RepeatMasker *.out* file had less than 2% Kimura 2-parameter distance from the consensus (we refer to this as the “recent-TE” dataset). This second analysis was required to effectively survey recent-TEs because *TEtranscript* analyses do not retain locus coordinates when returning the *.cntTable* output, which prevented us from being able to subsample recent-TEs directly from the primary inclusive analysis.
Genome assemblies and transcriptome annotations vary substantially in quality across species, also within mammals (e.g., MonDom5: 5,223 scaffolds, N50 = 108,014; mOrnAna1.p.v1: 305 scaffolds, N50 = 83,338,043). The presence in a genome of repeat sequences, such as TEs, represent a major challenge in genome assembly (Treangen, et al. 2011), as multimapping reads are a major cause of ambiguous calls, lower mapping scores, and ultimately scaffold incompleteness. One of the main pitfalls associated with traditional genome assembly techniques (i.e., second generation sequencing) is that the actual number of TEs in a genome tends to be underestimated, and this is particularly evident in low quality genome assemblies. However, while assembly quality can effect genomic estimates of the number of total and recent TEs, we don’t think it will substantially affect estimates of TE expression. This is because inferences of activity should not be highly dependent on the number of TEs annotated in the genome, but rather the existence (not the frequency) of sequences of these young TEs to provide a reference to map RNAseq to.

For each species, raw read counts for the entire transcriptome (genes and TEs) were normalized across tissues in *DESeq2* v1.20 ([Love, et al. 2014](#_ENREF_16)) after removing elements with less than 10 mapped reads across samples. Normalization was run independently on the total-TE dataset and on the recent-TE dataset; since gene expression estimates did not substantially differ between the two datasets (e.g., Supplementary file S2), we consistently used the normalized counts coming from the recent-TE dataset when analyzing gene expression levels. To examine patterns of within-species variation in expression profiles across tissues, we first applied a blind variance stabilizing transformation ([Anders and Huber 2010](#_ENREF_1)) to the entire count matrix, and used the resulting transformed data to calculate tissue-wise variance and evaluate patterns of expression using principal component analyses (PCAs).

To assess the relationships between expression levels of TEs and genes involved in TE negative regulatory mechanisms, we compared recent-TE expression levels to 5 sets of TE regulators: (i) genes participating in the PIWI:piRNA pathway ([Carbon, et al. 2009](#_ENREF_5); PIWI pathway hereafter); (ii) genes involved in the small RNA interference pathway ([Carbon, et al. 2009](#_ENREF_5); siRNA pathway); (iii) genes involved in transcriptional regulation of TEs (e.g., responsible for *de novo* DNA or histone methylation ([Hutchins and Pei 2015](#_ENREF_11); [Wylie, et al. 2016](#_ENREF_23)); (iv) other genes previously identified to negatively impact TE mobilization and/or insertion at the post-transcriptional level (e.g., Apobec; [Goodier 2016](#_ENREF_9)); and (v) the combined magnitude of the host response against TEs (all genes involved in negative TE regulation). The final gene dataset included a total of 79 genes, for which we recovered expression values of all annotated orthologues (Supplementary file S2).

Because of the heterogeneous nature of our data, we chose to perform between species comparisons using percentages of the total transcriptome following normalization of read counts to limit biases due to different methods of tissue processing, library preparation, sequencing technology and dataset quality ([Dunn, et al. 2018](#_ENREF_8); [Sudmant, et al. 2015](#_ENREF_21)). To test if proportions of TE-derived transcripts were significantly different across tissues, we performed a pairwise Wilcox test following a non-parametric Kruskal-Wallis rank sum test after assessing that the assumption of normality was not satisfied. To compare differences in patterns of gene expression levels across species and tissues, we calculated Z-score values of gene expression for each species using the *scale* function on log2 transformed normalized data in *R* (R [Team 2019](#_ENREF_22)). Z-scores were also used to perform hierarchical tissue clustering across species as part of the heatmap data visualization step generated using the *pheatmap* R package ([Kolde 2012](#_ENREF_13)) (distance method="euclidean"; hclust "complete" clustering). To assess differential gene expression in the testis and in the ovary compared to somatic tissues, we used log2 fold change values and corresponding adjusted p-values as calculated in *DESeq2*. Finally, to investigate relationships in TE and gene expression patters across vertebrates, we performed phylogenetic independent contrast (PIC) linear regressions and PCAs using the *phytools* package in *R* ([Revell 2012](#_ENREF_17)). Spearman rank correlation analyses were performed using the *rcorr* function in the Hmisc v4.2-0 R package ([Harrell 2019](#_ENREF_10)).

Anders S, Huber W 2010. Differential expression analysis for sequence count data. Genome Biology 11. doi: 10.1186/gb-2010-11-10-r106

FastQC: a quality control tool for high throughput sequence data [Internet]. 2010 [cited 2018. Available from: <http://www.bioinformatics.babraham.ac.uk/projects/fastqc>

Bolger AM, Lohse M, Usadel B 2014. Trimmomatic: a flexible trimmer for Illumina sequence data. Bioinformatics 30: 2114-2120. doi: 10.1093/bioinformatics/btu170

Bradnam KR, et al. 2013. Assemblathon 2: evaluating de novo methods of genome assembly in three vertebrate species. Gigascience 2: 10. doi: 10.1186/2047-217X-2-10

Carbon S, et al. 2009. AmiGO: online access to ontology and annotation data. Bioinformatics 25: 288-289. doi: 10.1093/bioinformatics/btn615

Casper J, et al. 2018. The UCSC Genome Browser database: 2018 update. Nucleic Acids Res 46: D762-D769. doi: 10.1093/nar/gkx1020

Dobin A, et al. 2013. STAR: ultrafast universal RNA-seq aligner. Bioinformatics 29: 15-21. doi: 10.1093/bioinformatics/bts635

Dunn CW, Zapata F, Munro C, Siebert S, Hejnol A 2018. Pairwise comparisons across species are problematic when analyzing functional genomic data. Proc Natl Acad Sci U S A 115: E409-E417. doi: 10.1073/pnas.1707515115

Goodier JL 2016. Restricting retrotransposons: a review. Mob DNA 7: 16. doi: 10.1186/s13100-016-0070-z

Hmisc V4.2-0 [Internet]. 2019. Available from: <https://CRAN.R-project.org/package=Hmisc>

Hutchins AP, Pei D 2015. Transposable elements at the center of the crossroads between embryogenesis, embryonic stem cells, reprogramming, and long non-coding RNAs. Sci Bull (Beijing) 60: 1722-1733. doi: 10.1007/s11434-015-0905-x

Jin Y, Tam OH, Paniagua E, Hammell M 2015. TEtranscripts: a package for including transposable elements in differential expression analysis of RNA-seq datasets. Bioinformatics 31: 3593-3599. doi: 10.1093/bioinformatics/btv422

Kolde R 2012. Pheatmap: pretty heatmaps. R package version 61: 915.

Kopylova E, Noe L, Touzet H 2012. SortMeRNA: fast and accurate filtering of ribosomal RNAs in metatranscriptomic data. Bioinformatics 28: 3211-3217. doi: 10.1093/bioinformatics/bts611

Leinonen R, Sugawara H, Shumway M, International Nucleotide Sequence Database C 2011. The sequence read archive. Nucleic Acids Res 39: D19-21. doi: 10.1093/nar/gkq1019

Love MI, Huber W, Anders S 2014. Moderated estimation of fold change and dispersion for RNA-seq data with DESeq2. Genome Biol 15: 550. doi: 10.1186/s13059-014-0550-8

Revell LJ 2012. phytools: an R package for phylogenetic comparative biology (and other things). Methods in Ecology and Evolution 3: 217-223. doi: 10.1111/j.2041-210X.2011.00169.x

Sayers EW, et al. 2019. Database resources of the National Center for Biotechnology Information. Nucleic Acids Res 47: D23-D28. doi: 10.1093/nar/gky1069

Schield DR, et al. 2019. The origins and evolution of chromosomes, dosage compensation, and mechanisms underlying venom regulation in snakes. Genome Res In Review.

RepeatMasker Open-4.0. 2013-2019. Available from: <http://www.repeatmasker.org>

Sudmant PH, Alexis MS, Burge CB 2015. Meta-analysis of RNA-seq expression data across species, tissues and studies. Genome Biol 16: 287. doi: 10.1186/s13059-015-0853-4

Sun YH, et al. 2017. Domestic chickens activate a piRNA defense against avian leukosis virus. Elife 6

Team RC. 2019. R: A language and environment for statistical computing. R Foundation for Statistical Computing, Vienna, Austria. 2013. In.

Treangen, TJ and Salzberg SL 2011. Repetitive DNA and next-generation sequencing: computational challenges and solutions. Nat Rev Genet 13(1): 36-46. doi 10.1038/nrg3117

Wylie A, Jones AE, Abrams JM 2016. p53 in the game of transposons. Bioessays 38: 1111-1116. doi: 10.1002/bies.201600115
